# Investigating the geochemical behavior and exploration potential of lithium in brines; a case study of Bam salt plug, Zagros Zone, southern Iran

**DOI:** 10.1038/s41598-023-48909-5

**Published:** 2023-12-07

**Authors:** Marziyeh Bazamad, Majid H. Tangestani, Sina Asadi, Michael Staubwasser

**Affiliations:** 1 Department of Earth Sciences, Faculty of Sciences, Shiraz University, Shiraz, 71454 Islamic Republic of Iran; 2 Department für Geowissenschaften, Mathematisch-Naturwissenschaftliche Fakultät, Institut für Geologie und Mineralogie, Universität zu Köln, Cologne, Germany

**Keywords:** Environmental sciences, Hydrology, Solid Earth sciences

## Abstract

Lithium (Li) is a scarce and technologically important element; the demand for which has recently increased due to its extensive consumption, particularly in manufacturing of Li-ion batteries, renewable energy, and electronics. Li is extracted from brines, pegmatite, and clay minerals; though extraction from brines is economically preferred. More than 200 salt plugs are in the Zagros Mountains which represent potential sources for Li exploration. This preliminary study collected first data on the abundance of Li in the salt plugs in southern Iran, and investigated Li distribution during evaporation of halite-producing brine ponds. The XRD analysis of powdered samples showed that gypsum and halite are the dominant solid phases in the ponds in which Li is concentrated in gypsum, while halite is depleted of Li. ICP-MS and ICP-OES analyses showed that Li in brines is concentrated during the evaporation by factors up to 28 with total contents up to 40 mg kg^‒1^. The Mg/Li ratio was higher than 70, which makes the brine unsuitable for conventional evaporation extraction techniques which require Mg/Li ratios of less than 6. Considering that 25 mg kg^‒1^ is a suitable concentration of Li for exploration purposes, the results of this study suggest that with the advancement of extraction techniques, the depletion of presently used high-grade Li reserves, the increasing demand for lithium, the need for extraction from diverse sources, and the exploration of new resources, the salt plug brines have an exploratory potential for Li in the future.

## Introduction

The importance of lithium (Li) as a strategic, critical, and technologically important element^1^ commodity stems from its high redox potential, excellent ionic conductivities, and low mass. Over the past few decades, the demand for lithium has significantly increased around the world^2^. This is mainly due to the important role of lithium in modern materials’ industry. Although the lithium market varies by location, the global end-use market is estimated as: batteries, 80%; ceramics and glass, 7%; lubricating fats, 4%; continuous mold melting powders, 2%; air handling, 1%; medical, 1%; and 5% for other use^3^. The demand for lithium and exploration of new resources has notably increased^4^ alongside advancements in battery technology and the accelerated penetration of electric vehicles^5,6^. This increased demand poses challenges to the global supply of lithium^7^.

Lithium is primarily sourced from three main resources^8^: (1) Brine solutions (e.g., salars, oilfield brines, geothermal systems) constitute over 60% of the identified reserves^4^. Important Li compounds obtained from brines are Li_2_CO_3_, LiCl, and LiOH. Lithium is also found in salt/brine mixtures lying beneath saline crusts as residues of ancient evaporated seas. Lithium-rich brine is the legacy of local volcanic activity transporting Li to the surface, where it can be leached by infiltrating waters^9^. Brine-based Li sources are found in various stages of geological development in Argentina, Bolivia, Chile, China, and the United States^10^. Around 25% of Li's world reserves are in the Salar de Atacama (SdA) (northeast Chile), which lies in the Pre-Andean depression to the west of the Altiplano-Puna^11^.

The study of hydrothermal fluids in brines has confirmed a thermal contribution^11^. The United States Department of Energy is funding a study on the possibility of extracting lithium from geothermal brines in the Salton Sea area of California. Initial estimates of lithium in the region indicate one of the world's most significant resources, but lithium extraction is not yet technologically or economically viable^12^.

(2) Pegmatites make 23% to 30% of the total identified Li reserves^13^ with typical Li-bearing minerals such as spodumene, petalite, and lepidolite. Small amounts of Li are also concentrated in minerals such as biotite, white mica, clinopyroxene, chlorite, glaucophane, and amphibole of metamorphic rocks with no commercial significance^14,15^.

(3) Clay deposits also present high lithium contents due to the low solubility of Li in seawater. Only 0.3% of the lithium supplied to seawater remains in solution while the remaining 99.7% is deposited as clay minerals^16^. Lithium deposits in sedimentary rocks are clays and lacustrine and account to less than 3% of the global Li resources. Lithium occurs in hectorite of clay and in jadarite of the lacustrine^17^. However, it is not extracted from these minerals, but could be a potential for the future^18^. Li^+^ has generally a positive correlation with Mg^2+^ and Al^3+^, and can be included into the crystal structure of clay minerals^19^ in which, Li^+^ substitutes for Mg^2+^ in the octahedral layer^20^. In general, lithium extraction from brines is preferable because of lower costs relative to pegmatites and clays^21,22^.

An additional potential of lithium resources that has received little attention are salt plugs. Salt plugs are dome-shaped geological structures that have been uplifted in the form of circular or elliptical bumps. The uplift is usually triggered by sudden shakes from earthquakes or tectonic forces^23^. Thick salt deposits are known in several tectonic settings: (1) cratonic basins (Zechstein, Pricaspian); (2) syn-rift basins (Hormuz Zagros, Morocco, Maritime Canada, Sverdrup); (3) late syn-rift or post-rift passive margins (Gulf of Mexico, South Atlantic) and (4) continental collision zones and foreland basins (Gachsaran Zagros, Messinian, Great Kavir, Paradox).

Southern Iran features over 200 salt plugs, found both onshore and offshore^24^. They constitute the Infra-Cambrian Hormuz Salt series. In places, where the plugs are free of deformations, the salt layers are covered by more than 6 km of sedimentary rocks^25^. Possible reasons for significant lithium enrichment in brines within closed reservoirs include the occurrence of Li-rich rocks or clay minerals, hydrothermal activity, arid climate, and tectonic subsidence^26^. Under the conditions of formation of salt plugs that lead to Sabkha and Playa environments, formation of clay minerals and evaporates, as well as felsic igneous rocks, Li can be enriched in salt plugs.

Increasing demand for clean energy and the growing adoption of electric vehicles and renewable energy sources have significantly increased global demand for lithium, making it a strategic resource for transition to a low-carbon economy^27^. Given this increased demand, the world should be concerned about future lithium supplies. The growing demand for lithium has sparked interest in extracting it from various sources and exploring new resources.

However, no comprehensive research has so far been conducted on Li enrichment in salt plug brines. The objective of this research was to investigate the potential of salt plugs as a source of lithium. Particularly, we were interested in studying the concentration of Lithium and other salt components during evaporation in a halite production site of the Bam salt plug, southern Iran. We assumed that the interplay of increase in concentration of lithium in the brines caused by the evaporation and the precipitation of halite has an impact on the Li concentrations in the brines and in salt deposits in the production site; a process we called “brine evolution”. Moreover, we have attempted to determine the source of the Li in the brines from the mineral deposits in the salt plug. This study is a preliminary investigation to examine whether the salt plugs in southern Iran could be considered as a potential for lithium at the future.

## Materials and methods

### Study area

**Figure 1:**
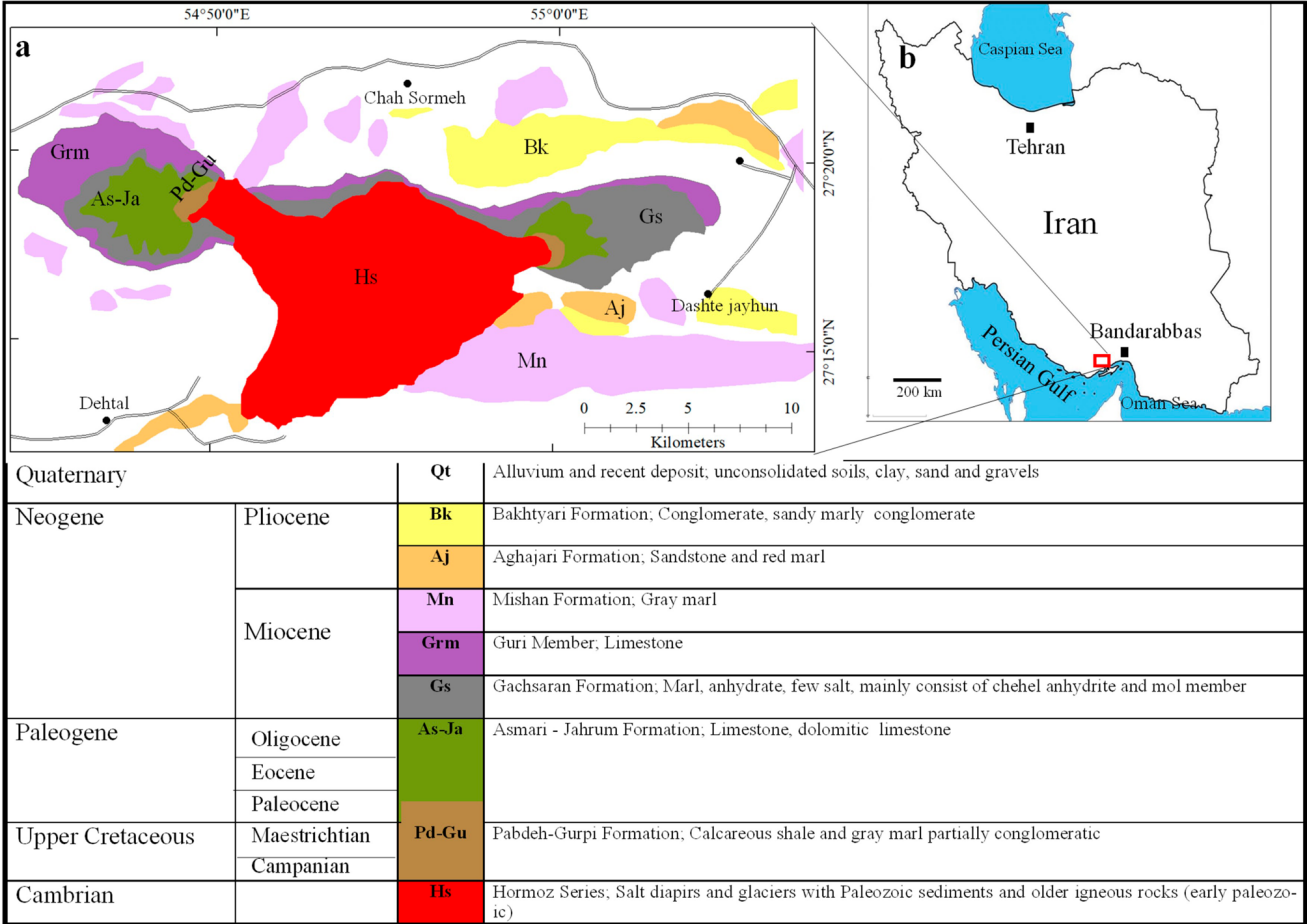
**(a)** The geological map of the study area (the map generated through ArcGIS^37^). **(b)** The location of study area in Iran.

The study area is located in an arid to semiarid region of the Hormozgan Province, southern Iran (Fig. 1). This area is part of the Zagros Folded Belt (ZFB) which is a result of the Neogene Collision between the Afro-Arabian and Iranian plates^28^. The Zagros orogenic belt covers an area of about 700,000 square kilometers in the central part of the Alpine-Himalayan orogenic system and is one of the largest areas that has undergone transpersonal deformation^29^. The belt is formed by the Neo-Tethys subduction with NE-dipping under the Iranian subcontinent in the Jurassic to Paleogene. This was followed by the Neogene oblique collision of the central Iranian microcontinent with the Arabian plateau^30^, where post-collision crustal shortening is still effective^31^.

The Hormuz Series, also called Hormuz Evaporates, is a typical sequence of evaporitic–volcanic rocks exposed in the form of emerging salt plugs or salt diapirs within the anticlines of the Zagros Belt^32^. These series were deposited during the Infra-Cambrian in the Proto-Tethys subsiding rift basins along the Middle Eastern edge of Gondwana^33^. The Hormuz salt formed in a rectangular basin bounded by deep crustal faults, along the edge of the Arabian plate's continental rift^32^. Deposition and initial movement of the Hormuz salt were controlled by rifting events that occurred in the Lower Paleozoic^34^. The Hormuz series is characterized by igneous and sedimentary rocks that suffered multiple geological episodes in their^35^ and consists of four lithological units^25^ including: (1) salt beds with fine intercalations of marl, tuff, sulfide mineralization, limestone, and iron oxides; (2) intercalations of finely laminated algal limestone with alternating anhydrite, marl, ignimbrite, tuff, and ironstone components; (3) laminated black fetid algal limestone; (4) alternations of tuff, sandstone, and marl with some intercalations of anhydrite and black algal limestone.

The Bam salt plug is situated between 54°50′ to 55°01′ East Longitudes and 27°12′ to 27°18′ North Latitudes, approximately 120 km west of Bandar Abbas, in Hormuzgan Province, Iran.

It is situated at the central part of the Kuh-e-Abad Anticline (Fig. 1) that consists of the Hormuz, Pabdeh, Gurpi, Asmari, Jahrom, Gachsaran, Mishan, Agha Jari, and Bakhtyari Formations. The activity of this plug during the Middle Miocene is supported by the condensed profiles of the Agha Jari and Mishan Formations^36,37^.

**Figure 2:**
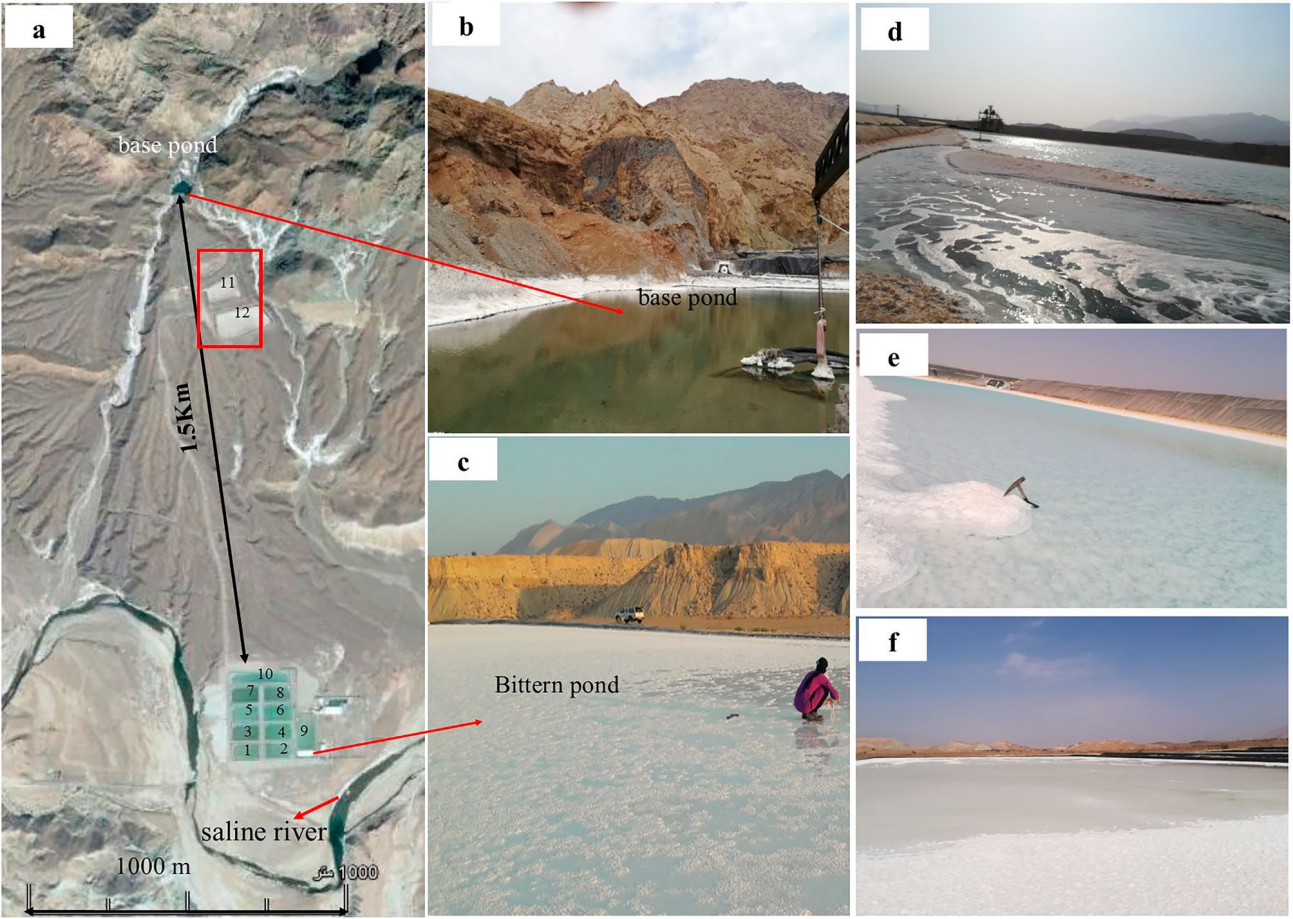
Sampling sites of liquids from the ponds. **(a)** Location of salt ponds in the study area, **(b)** base pond, **(c)** Bittern pond, **(d)** t1, **(e)** t3, **(f)** t4.

**Figure 3:**
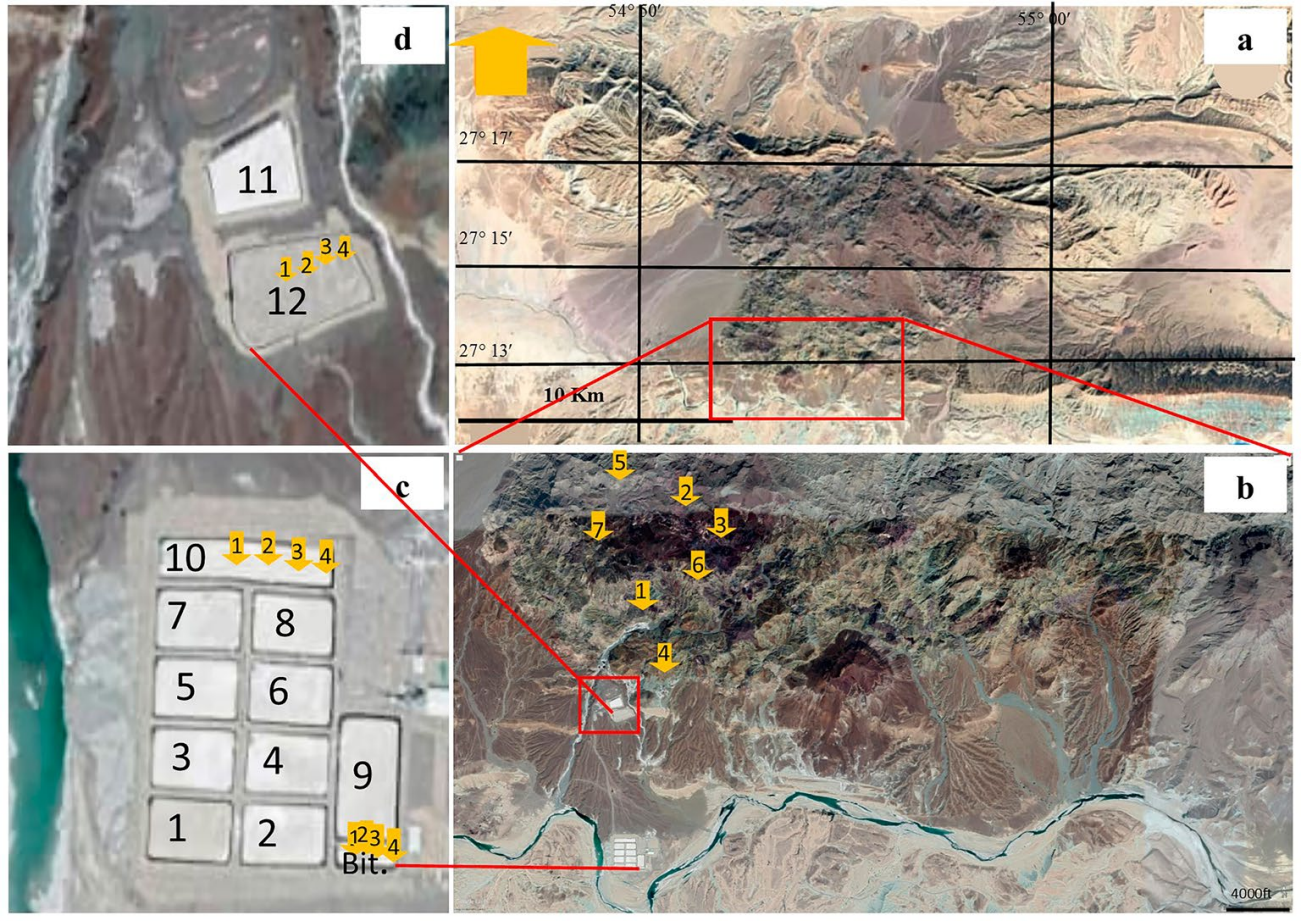
**(a)** Study area in the Bam Salt plug shown in satellite image; **(b)** The sampling points for solid samples 1 to 7, **(c)** The location of solid samples 10-1 to 10-4 in pond 10 and Bit-1 to Bit-4 in the Bittern pond, **(d)** the sampling points for solid samples 12-1 to 12-4 in pond 12. Sampling points are represented by yellow arrows.

The Bam salt plug is a big salt diapir with large salt glaciers. It has a trapezoidal to triangular shape with maximum length of 16 km and maximum width of 9 km. The plug materials are partially covered by Upper Miocene sediments^38^ and karstic phenomena are developed at the eastern margins of the plug. Few NE-SW, W-E, and NW–SE trending fractures have dissected the plug. The plug activity can be considered finished owing to the general plug morphology^38^. There is a halite production site at the southern limb of the Bam salt plug (Figs. 2, 3).

### Brine sampling

Thirteen salt precipitation artificial ponds for extraction of halite are active at the study area (Fig. 2). A base pond, 1.5 km upstream the production ponds (Fig. 2a), is fed by a stream which runs through the salt plug. Following the evaporation of approximately half of the brine and precipitation and recovery of the halite, the remaining brine of the ponds is transferred to the Bittern pond. The final brine from the Bittern pond is transported into the adjacent saline river (Fig. 2b). The brine initial composition of the ponds 1 to 10 is the same as they are all fed from the base pond at the same time. Therefore, only pond number 10, which was larger than the others, was sampled.

The samples were taken at regular intervals to investigate the evolution of brine over time during evaporation. Because the ponds’ water intake cycle is once every two weeks, samples were collected five times over a two-week period after the intake; thus every 3 days (t1 to t5, Table 1, Fig. 2d–f), from the surface of the ponds and from the area lying 10 to 20 cm below the surface, which is called “interstitial”^39^. The expected concentration/composition changes over this period were called “brine evolution” (Table 1). Three samples were also collected from the Bittern pond over a two-week period. Over time, the brine evaporates more and its volume decreases. It is possible to decrease the volume of brine and to increase evaporation and thus to investigate the evolution of brine by comparing its volume in different times. The observed volume of brine decreased from the base pond during the times t1 t2, t3, t4 and t5, respectively. Thus t5 and bit-C (Table 1) were collected when halite was precipitated and most of the ponds were solidified (Fig. 2c).

**Table 1:** Numbering and naming of the brine samples—“the brine evolution”.

Entry no.	Brine evolution step	Sampling place	Sample name	Type of brine	Sampling date
1	1	Base pond	bp	Surface	2019.08.27
2	2	Pond 10	t1	Surface	2019.08.30
3	3		t2	Surface	2019.09.02
4	4		t3	Surface	2019.09.05
5			t3-I	Interstitial	2019.09.05
6	5		t4	Surface	2019.09.08
7			t4-I	Interstitial	2019.09.08
8	6		t5	Surface	2019.09.12
9	1	Bittern pond	bit-A	Surface	2019.08.30
10			bit-A-I	Interstitial	2019.08.30
11	2		bit-B	Surface	2019.09.05
12			bit-B-I	Interstitial	2019.09.05
13	3		bit-C	Surface	2019.09.12
14			bit-C-I	Interstitial	2019.09.12

The liquid samples (Table 1) were collected and were filtered by 0.4 µm polypropylene syringe, and the duplicate samples were then stored in polyethylene bottles. Samples for anion analyses were stored in non-acidified bottles, while samples for cation analyses were acidified with 0.1 N HCl, and were stored in acid-washed polyethylene bottles. Concentrations of Li, Na, K, Mg, B, Sr, S, and Cl were determined in all samples by inductively coupled plasma optical emission spectroscopy (ICP-OES; Spectro Arcos, AMETEC, Germany) at the Geological Institute, Department of Geosciences, University of Cologne. Cl was analyzed with an attached auto sampler from ESI and was measured at 134.724 nm, with a range of 1–16 mg/l (DL calc by 10 × Stdev of blank mean, added to mean value of blank). For Quality check, the Battle02 was certified as reference material, and an internal reference material called ATA-Salz-Int, (also a Merck VI Multielement Standard) was used to check the calibration. (The operational parameters for ICP-OES analyses are defined in Supplementary Table S1).

The major anions and cations including Li^+^, Ca^2+^, Mg^2+^, Na^+^, K^+^, HCO_3_^‒^, SO_4_^2‒^, and Cl^‒^ of sample number 2 from pond 10 and the Bittern pond were measured in the hydrochemical Lab of Shiraz University for the Piper diagrams. Ca^2+^ and Mg^2+^ were analyzed by titration with EDTA (ethylene diamine tetra acetic), using Murexide and Erichrom Black-T as indicators. HCO_3_^‒^ concentrations were determined by titration with HCl using methyl orange as an indicator. Li^+^, Na^+^ and K^+^ contents were measured by the standard F-AAS method (BWB Flame Photometer, BWB Technologies, Newbury, Berkshire, UK), Cl^−^ concentrations by the Mohr method, and SO_4_^2‒^ by turbidity method.

### Solid samples (rocks and pond deposits)

After studying 60 rock samples in a preliminary field survey, twenty microscopic thin sections from lithological units of the salt dome were prepared at the Shiraz University, Shiraz, Iran, and were studied using a polarizing microscope at the Geological Institute, University of Cologne, Germany. Out of them, seven solid samples were selected from rock outcrops (samples 1 to 7, Fig. 3b) for ICP-MS analysis along with 12 samples collected from deposits of ponds 10 (samples 10-1 to 10-4) and 12 (samples 12-1 to 12-4) and from Bittern pond (samples Bit-1 to Bit-4). All solid samples were analyzed for 59 main and trace elements using inductively coupled plasma mass spectrometry using ICP-MS, Perkin Elmer Sciex ELAN 9000 model instrument (Perkin-Elmer, Waltham MA, USA) at Iran Mineral Processing Research Center (IMPRC), Karaj, Iran. The samples were finely ground using an agate mortar to pass a 75 µm mesh. Typically, 100 mg of each sample was digested sonicating them in 0.3 M HNO_3_, HCl, 5% HF and H_2_O_2_, and finally dissolved in distilled water under sonication.

Deposit samples from ponds 10 and 12, and the Bittern pond were powdered to < 74 µm using a PM 400 agate mill (Retsch, Hahn, Germany) and were analyzed for mineral phases using a powder X-ray diffraction (PXRD) instrument at the Institute for Inorganic Chemistry, Department of Chemistry, University of Cologne. The data were collected at room temperature on a Huber G670 diffractometer (Huber, Rimsting, Germany) equipped with a germanium monochromator, Cu *K*_α1_ radiation and an imaging plate detector. For the purposes of measurements, the materials were placed as flat samples between two foils (foil reflections at 2θ ≈ 21.5°and 2θ ≈ 23.7°). Typical recording times were 30 min. The software X'Pert High Score Plus (PANalytical X’Pert Powder Pro, Malvern Panalytical, Almelo, Netherlands) was used to analyze the XRD output data.

## Results and discussion

### Petrography

**Figure 4:**
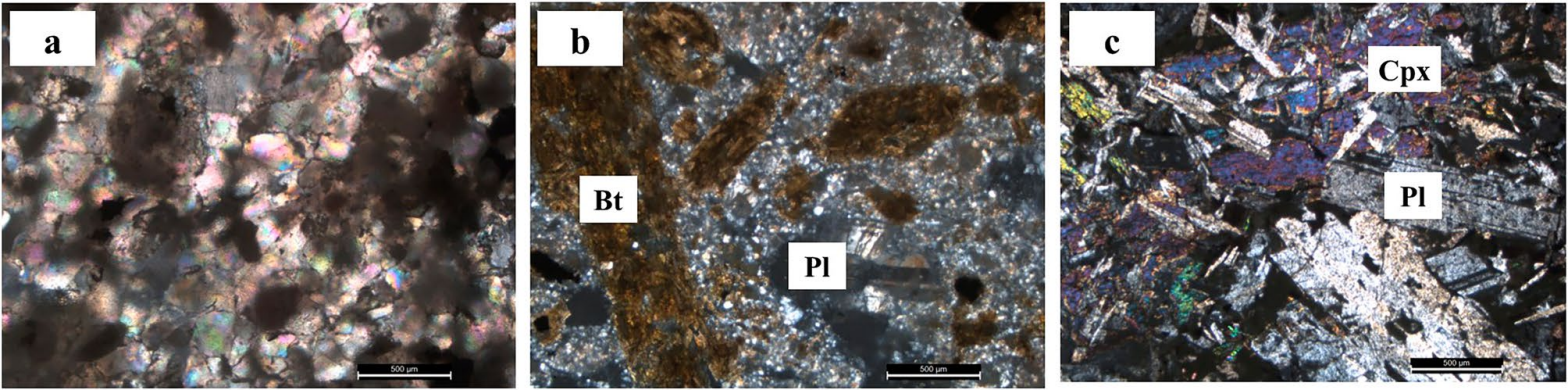
Microscopic thin sections of **(a)** shale, **(b)** rhyolite, **(c)** gabbro, *Bt* biotite, *Pl* plagioclase, and *Cpx* clinopyroxene.

Detailed petrographic studies showed the occurrence of chemogenic and clastic sedimentary rocks as well as magmatic units in the Bam salt plug. The sedimentary rocks include red, purple-red, and grayish-red shale to siltstone, grayish to reddish sandstone, marl, limestone, sandy and dolomitic limestone, dolomite, quartzite, chert, grayish-brown to red salt, gypsum, red shale of ochre nature, and limonitized and hematitized rocks (Supplementary Fig. S1). The red color of salt is due to the presence of hematite, and the grayish-brown color is caused by enclaves of shale and sandstone. The magmatic rocks are generally altered outcrops of gabbro, diorite, granodiorite, carbonatized tonalite, andesite, basalt, and rhyolite (Supplementary Fig. S2). Spherical spots of shale are scattered at the area and show a pronounced conchoidal fracture. They predominantly consist of clay matrix with very small grains of quartz, feldspar, muscovite, and chlorite (Fig. 4a).

Rhyolites include quartz, biotite, plagioclase, and clay minerals as the major and minor phases (Fig. 4b). Granite (rhyolite) is a common igneous rock that can concentrate lithium-bearing minerals, particularly if lithium incompatibility causes magmatic differentiation late in crystallization process and enrichment in final stages of magma solidification.

Gabbro is composed of plagioclase and clinopyroxene with crystal sizes ranging from 200 μm to 8 mm (Fig. 4c), amphibole and epidote as well as accessory minerals including olivine, chlorite, apatite, actinolite, albite, calcite, and opaque minerals. The anhedral to subhedral opaque minerals are occurring in a size range of 120 to 250 μm.

As evaporation progressed, white salt will precipitate in the brines. The principles behind the formation of evaporate deposits are relatively simple in theory, although actual deposits can be complex and exhibit a large variety of salt minerals and paragenetic sequences. As sea water or brine evaporates and water vapor is removed into the atmosphere, the salinity (i.e. the total content of dissolved salts in solution) of the residual solution increases and individual salts precipitate as their solubility limits are reached. The order or sequence of precipitation reflects the scale of increasing solubility at a given temperature, such that the salts with the lower solubility precipitate first, followed by salts with higher solubility. Order of precipitation of minerals of sea water are calcite (CaCO_3_) gypsum/anhydrite (CaSO_4_/CaSO_4_.2H_2_O), halite (NaCl), epsomite (MgSO_4_.7H_2_O), kainite (KMgClSO_4_.3H_2_O), sylvite (KCl), MgCl_2_, borates, and sulfates^40^. Powder XRD (PXRD) analysis of sediments collected from ponds 10 and 12 and the Bittern Pond showed gypsum and halite as the major phases (Supplementary Fig. S3). Gypsum precipitates in early stages of evaporation which leads to the depletion of Ca^2+^ in the brine. The distribution of precipitated sediments in the ponds provides a general zoning in which, based on the PXRD results, gypsum and halite are the solid phases precipitated as concentric haloes from the margin to the center of the ponds, sequentially.

### Geochemistry

**Figure 5:**
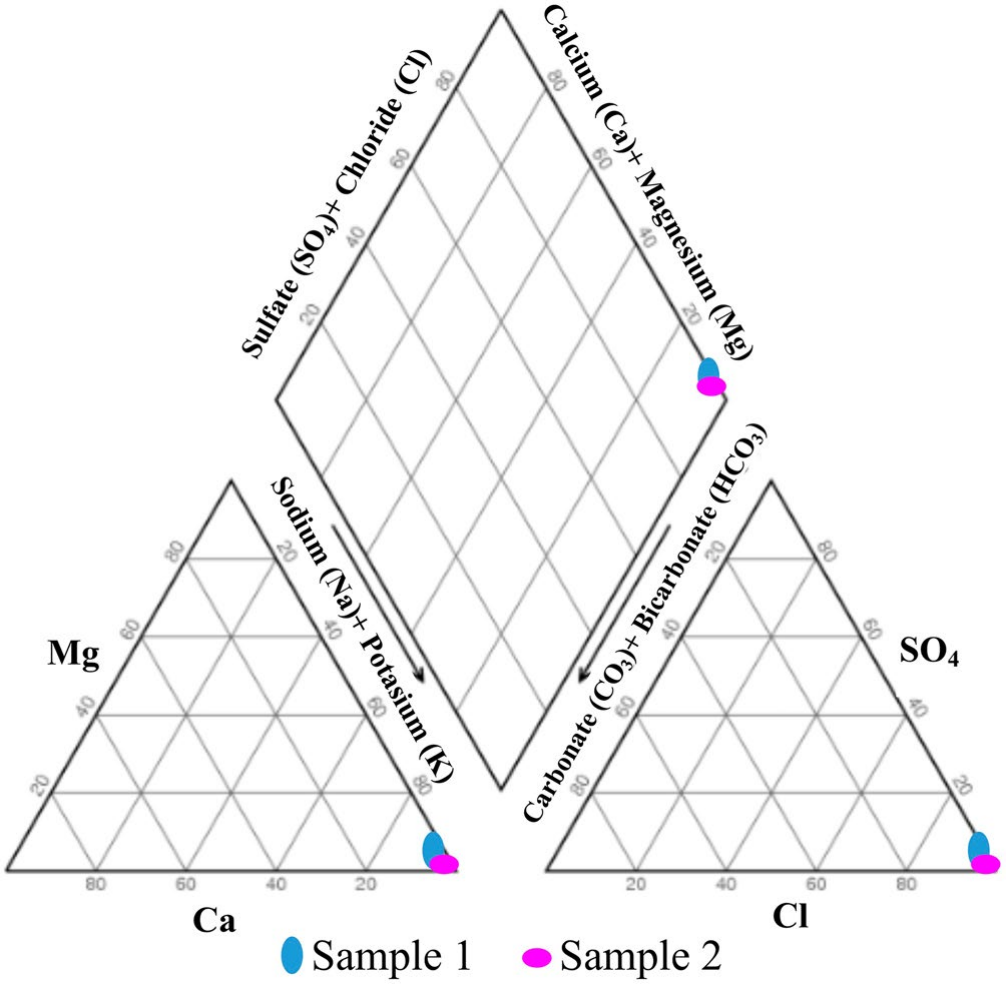
Piper diagrams of anions and cations of the Bam salt plug brine. Sample 1: brine of pond 10; Sample 2: Bittern brine.

Generally, the chemical composition of brines determines the deposited phases and the order of their deposition^41^. The geochemical evolution of a water body can be understood by plotting the concentrations of major cations and anions on a trilinear Piper diagram. This diagram divides water into her four basic types, depending on the placement of the diamonds near her four corners. The water visible at the top of the diamond is rich in Ca^2+^  + Mg^2+^ and Cl^−^ + SO_4_^2−^ and creates an area of permanent hardness. The water near the left corner is rich in Ca^2+^  + Mg^2+^ and HCO_3_^−^ and is a temporary hard water region. The water applied to the lower corner of the diamond is mainly composed of alkaline carbonates (Na^+^  + K^+^ and HCO_3_^–^ + CO_3_^–^), while the water near the right side of the diamond may be considered saline (Na^+^  + K^+^ and Cl^−^ + SO_4_^2−^)^42^. The cations plotted in the diagram are showing the dominance of Na^+^ or K^+^ type and in anion plot it is clearly seen that Cl^−^ is dominant. Thus, according to the Piper diagram, the brine of the Bam ponds belongs to the sodium chloride type (Fig. 5).

**Figure 6:**
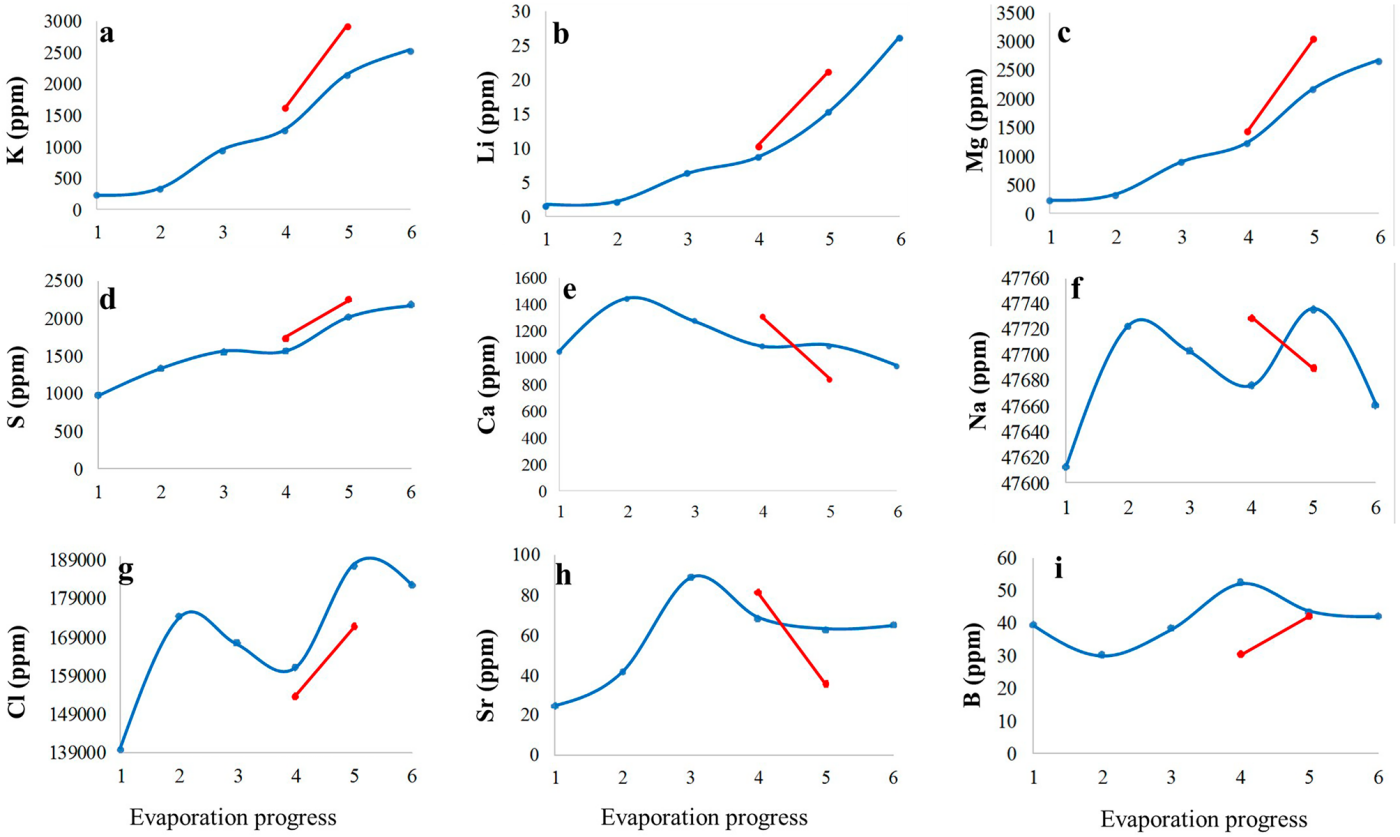
Distribution of elements in ponds during the brine evolution. Blue: surface brines, red: interstitial brines.

**Figure 7:**
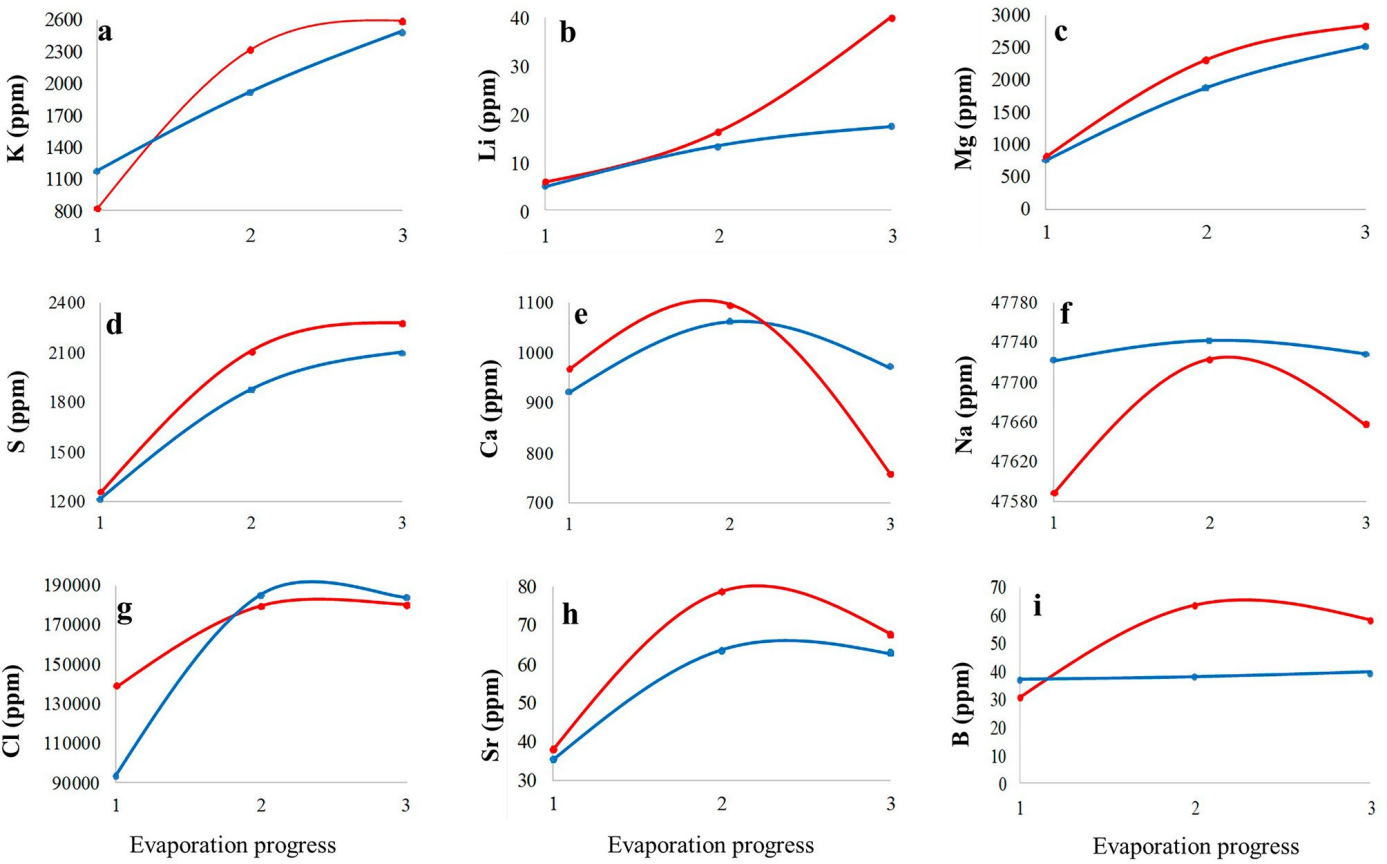
Distribution of elements in Bittern pond during the brine evolution. Blue: surface brines, red: interstitial brines.

The effect of evaporation on element concentrations in the ponds, what we call “brine evolution”, can be deduced from Table 2. The abundances of K, Li, Mg, and S increase in brine during evaporation (Figs. 6a–d, 7a–d). Meanwhile, the concentration of lithium increases from 1.4 mg kg^‒1^ in the surface brine of the base pond (bp) to 26 mg kg^‒1^ in the surface brine (t5), and from 5.02 mg kg^‒1^ in bit-A to 40 mg kg^‒1^ in the interstitial brine of the Bittern Pond (bit-C-I).

**Table 2:** Concentrations (mg kg^‒1^) of elements in brines.

Brine evolution	Sample name	Brine type	Li	B	Sr	K	Mg	Ca	S	Na	Cl
1	Base pond		1.4	39.47	24.76	219	209	1042	973	47,612	139,875
	bp	Surface									
	Pond 10										
2	t1	Surface	2.01	30.25	41.93	307	301	1439	1337	47,722	174,425
3	t2	Surface	6.27	38.47	88.65	916	878	1273	1545	47,702	167,443
4	t3	Surface	8.62	52.48	68.19	1240	1203	1080	1558	47,675	161,184
	t3-I	interstitial	10.15	30.59	81.79	1608	1430	1305	1741	47,729	153,737
5	t4	Surface	15.16	43.43	62.31	2127	2158	1085	2018	47,734	187,346
	t4-I	interstitial	20.97	42.18	35.75	2905	3042	833	2266	47,690	171,988
6	t5	Surface	26.06	42.12	65.31	2512	2640	931	2185	47,660	182,350
	Bittern pond										
1	bit-A	Surface	5.02	6.75	35.58	1176	761	921	1213	47,722	92,981
	bit-A-I	interstitial	5.86	30.4	38.1	828	803	968	1259	47,588	139,250
2	bit-B	Surface	13.2	37.61	63.59	1911	1860	1061	1873	47,741	184,455
	bit-B-I	interstitial	16.22	63.5	78.65	2320	2301	1094	2107	47,722	179,460
3	bit-C	Surface	17.33	39.03	67.72	2475	2502	971	2090	47,727	183,783
	bit-C-I	interstitial	40.01	58.32	63.17	2582	2832	758	2274	47,658	180,155

An increase of Ca is observed from the base pond to sample t1, while in the further course of the brine evolution the Ca content decreased to 931 mg kg^‒1^ in sample t5. A similar evolution is found in the bittern pond with an overall decrease from 921 mg kg^‒1^ in bit-A to 758 mg kg^‒1^ in interstitial brine of sample bit-C-I (Figs. 6e, 7e). The Ca diagram (Fig. 6e), representing the brine evaporation process, shows that Ca is enriched in the early stages of evaporation, and then, it is depleted. The S content increases through the evolution of brine. Thus the depletion of Ca is probably corresponded to the precipitation of CaSO_4_ which is supported by PXRD analysis. A similar trend to Ca is found for Sr, although retarded. Following marked increases from base pond to sample t2 (values of approximating 90 mg kg^‒1^), Sr is subsequently depleted, likely due to the precipitation of SrSO_4_. However, we did not find SrSO_4_ in the PXRD, so its precipitation remains an assumption. Moreover, when looking at the course of the Na and Cl contents, a general decrease was observed following marked initial increases. Overall, Na varies from 47,734 mg kg^‒1^ in t4 sample to 47,660 mg kg^‒1^ in t5 sample, and between 47,741 mg kg^‒1^ to 47,588 mg kg^‒1^ in the Bittern Pond. Cl varies from 139,875 mg kg^‒1^ to 187,346 mg kg^‒1^ (Fig. 6f, g) and from 92,981 to 184,455 mg kg^‒1^ in the Bittern Pond (Fig. 7f, g). Finally, there was no trend in abundancies of B (Figs. 6h–i, 7h–i) and Si (not shown). We assume that borosilicates are abundant as particular matter and thus are not subject to concentration or precipitation.

Distribution of immobile trace elements in solid samples were also studied to probe the tectonic evolution leading the salt plug emerge, and the formation of brines (Supplementary Table S2, locations of the samples are shown in Fig. 3). In contrast to the rock material, most elements are completely or nearly completely depleted in salts, with the exception of the alkali metals, the alkaline earth elements, As, Cu, Cr, Cd, Ga, Ni, and Te. Ga, Cu, and the alkaline earth elements are depleted in the salts compared with the rock samples but they still are abundant in sizeable quantities. The heavy alkaline metals Sr and Ba show quite high contents in the salts reaching up to 175 mg kg^‒1^ in sample 10-4 for Sr and 113 mg kg^‒1^ in Bit-3, while the lighter elements Mg and Ca are largely depleted in the salts.

A closer look on the alkali metals show that Li is depleted in most salt samples, excepting the sample 12-4 from the evaporated pond 12 that reaches 30 mg kg^‒1^. The highest Li content in rock samples was found in andesite, rhyolite, and shale. For Na, a marked enrichment by a factor of up to 10 was found in the salts. Potassium is generally depleted in the salts but still reaches up to 0.7%. The salts contain significant amounts of Rb, as evidenced by sample 12-4 from pond 12 containing up to 35 mg kg^‒1^. Cesium has a rather constant value ranging from 4.1 to 7.6 mg kg^‒1^ in rocks and 4.5 mg kg^‒1^ to 9.3 mg kg^‒1^ in salts.

We focused on trace element correlation on rhyolite because it is the dominant rock type. Based on the Co vs. Th^43^ and Ta/Yb vs. Th/Yb diagrams^44^ (Supplementary Figs. S4a, S4b), the Bam rhyolite was categorized in high-K calc-alkaline and shoshonitic class. On the basis of the Pearce diagrams^45^ of Rb vs. Y + Nb, Ta vs. Yb, Rb vs. Y + Nb, and Th/Yb vs. Nb/Yb, the Bam rhyolite is plotted in the “volcanic arc” domain (Supplementary Fig. S4c–f), and shows the characteristics of volcanic rocks generated in active continental margins. The same can be concluded from the^46^ diagrams of Th/Yb vs. Ta/Yb, Th vs. Ta, Th/Hf vs. Ta/Hf, and Th/Ta vs. Yb (Supplementary Fig. S5), which is completely consistent with previous studies^32,34^.

### Correlation coefficients

**Figure 8:**
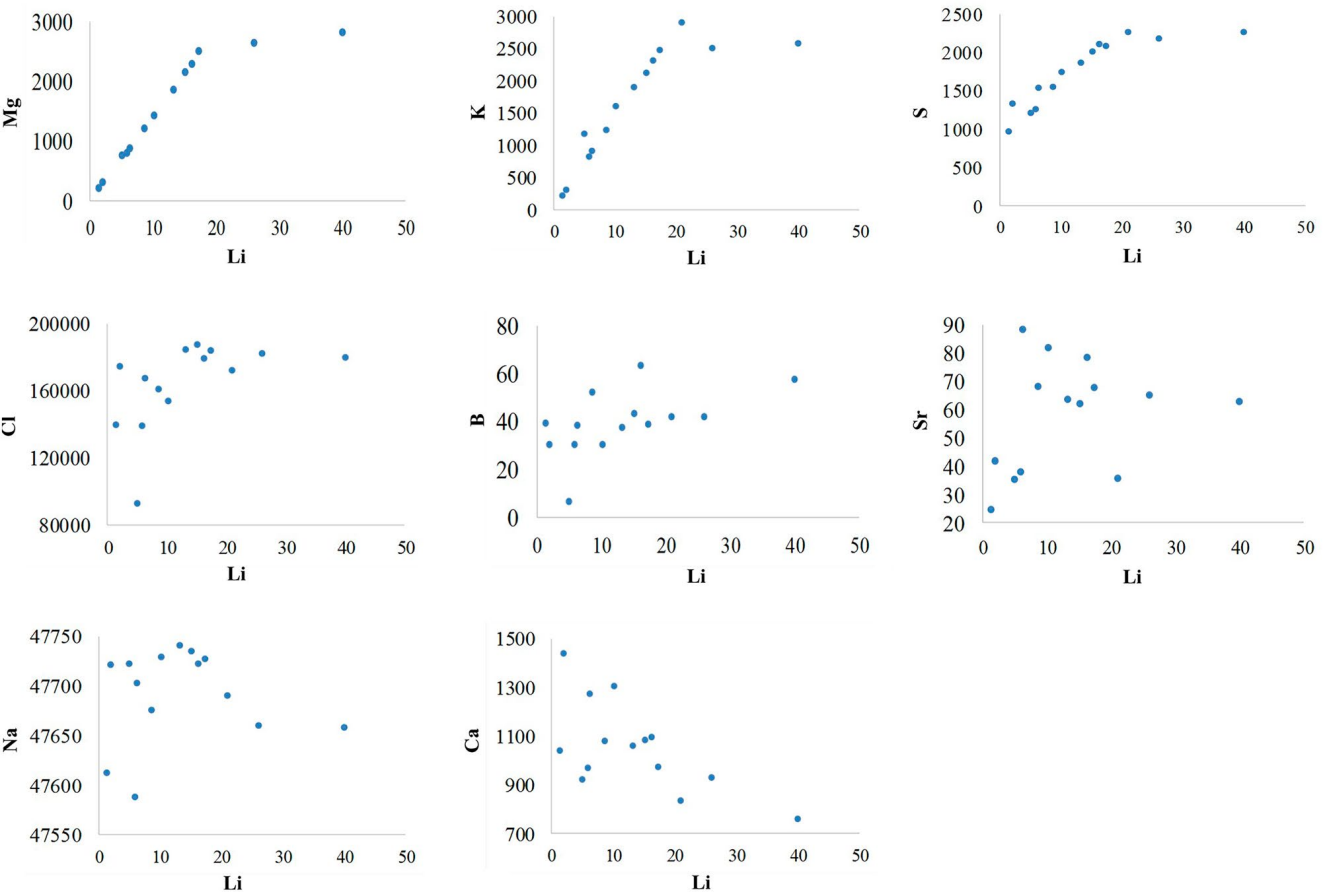
Scatter diagrams of Mg, K, S Cl, B, Sr, Na and Ca against Li (mg kg^‒1^) calculated from Supplementary Table S2.

To get more insight into the brine evolution, we calculated the relative concentration factors of Li from different brines (Fig. 8). According to the scatter diagrams, Li shows positive correlations with Mg, B, K, Cl, and S during brine evolution. It also shows negative correlations with Ca and Na, and no significant correlation with Sr. In contrast to Na, K, Cl, Mg, and Ca, B is assumed to be a conservative element in chemical precipitation which means that B either does not precipitate or shows poor precipitation^47^ which corresponds to our earlier assumptions. Therefore, the relative concentration factor was calculated based on the B concentration.

**Figure 9:**
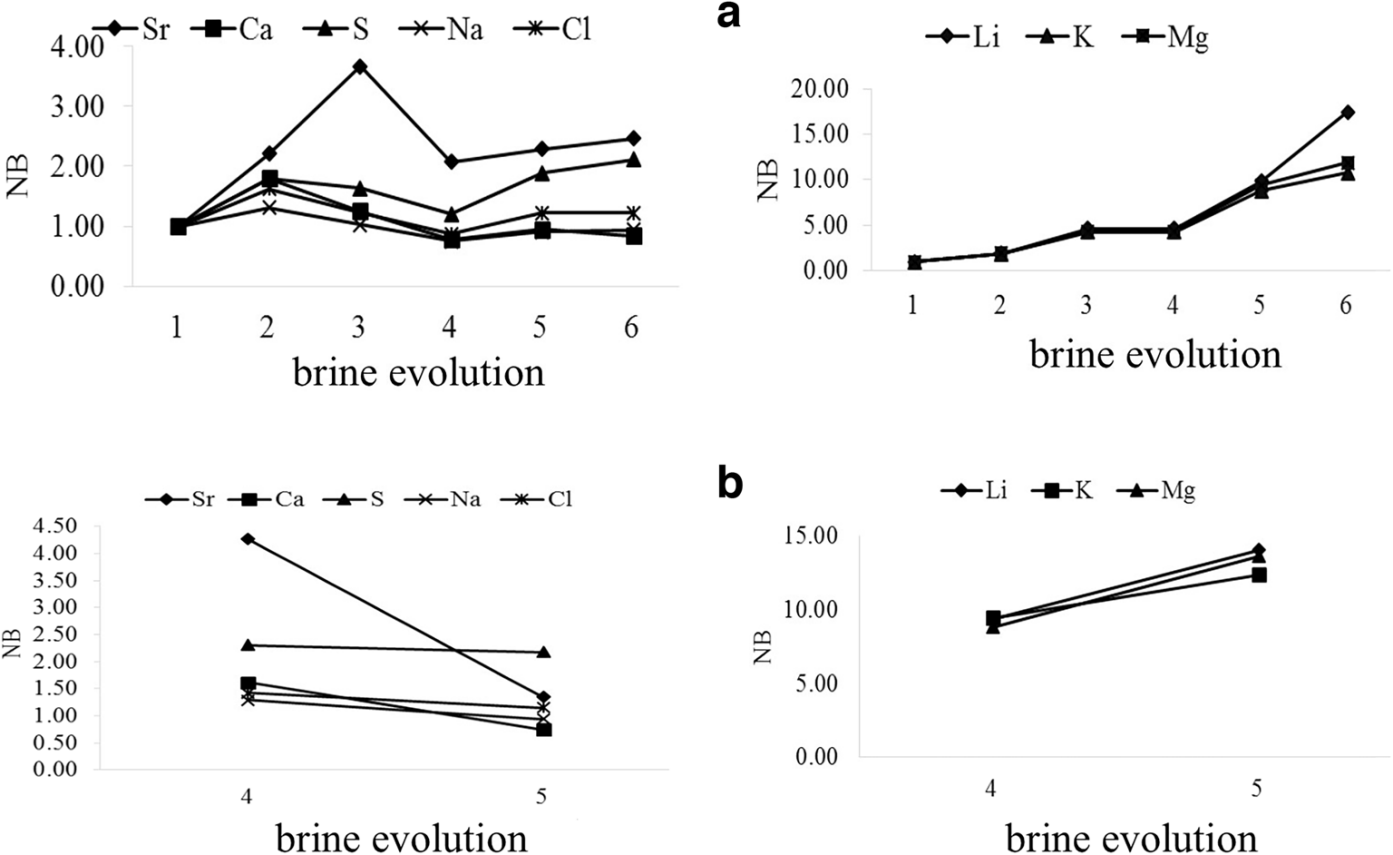
Boron-normalized concentration ratios (NB) during the evaporation of the brine in the ponds. **(a)** Surface brines, **(b)** interstitial brines.

**Figure 10:**
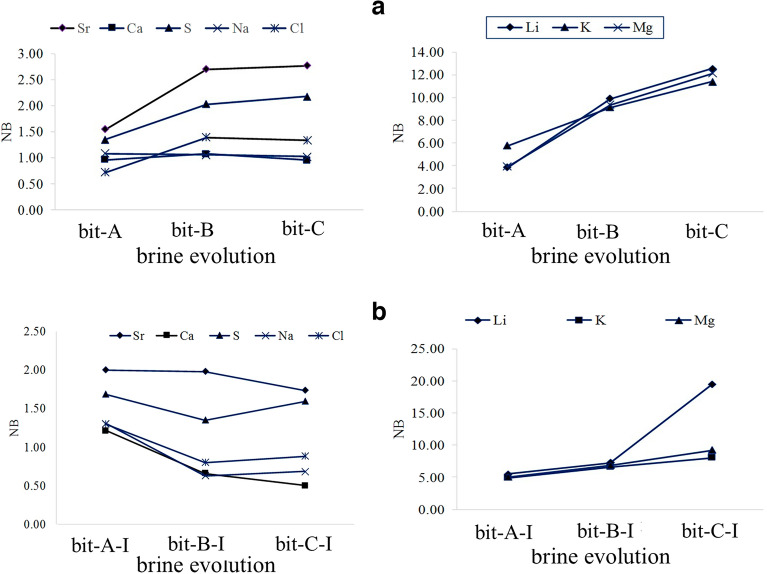
Boron-normalized concentration ratios (N_B_) during the evaporation of the brine in Bittern pond. **(a)** Surface brines, **(b)** interstitial brines.

The Boron-normalized concentration of each element as a result of water loss due to evaporation was calculated using Eq. (1):

1 $$ {\text{N}}_{{\text{B}}} = \, \left( {{\text{M}}/{\text{B}}} \right)_{{\text{n}}} /\left( {{\text{M}}/{\text{B}}} \right)_{{\text{i}}} $$

where; (M/B)_n_ is the concentration ratios of an element and Li at a certain stage of evaporation, and (M/B)_i_ represents the initial ratio. If an element does not precipitate, its NB is expected to be unity (1.0), but if the element precipitates, the parameter is lower than 1^48^. The changes of N_B_ pattern for the targeted elements are shown in Figs. 9 and 10. As a result of evaporation in the halite-bearing ponds, the average of NB for Na, Ca, Li, K, Mg, and Cl were 1, 1.1, 6.6, 5.15, 5.5, and 1.2, respectively, showing a marked enrichment for Li, Mg, and K. The values in interstitial brine of the Bittern pond were 0.9, 0.4, 10.7, 6.5, 7, and 1, respectively (Supplementary Table S3), with the highest value for Li, again followed by Mg and K. This indicates that during the Bam evaporation, the loss of cationic species is approximately proportional to the loss of anionic species, and elements Li, K, and Mg, which remain soluble in the brine and do not precipitate during the final stage of evaporation.

### Development of Li concentration in brines

As expected, the concentration of Li increased in the brines during the evaporation process as well as Mg and K, while the concentration of Na stayed rather constant (Table 2). The averaged concentration of Li in ponds was 15.5 mg kg^‒1^ in interstitial brine, while its average value at the surface brines of ponds was 11.6 mg kg^‒1^. The average concentration of Li in interstitial brine and surface brine of the Bittern Pond were 20.7 mg kg^‒1^ and 11.8 mg kg^‒1^, respectively. The concentration of Li in interstitial brines reaches up to 28 times that of the base pond. The concentration of Na in the brines is so high that it probably hampers KCl precipitation resulting in increased concentrations of K. This in turn, prevents the precipitation of Mg, and subsequently, impedes the precipitation of Li^48^. The final result of this cycle is increment of Li concentration in the brine through evaporation.

As the evaporation and precipitation of gypsum and halite proceed, the elements Ca and Na decrease in the brine, while incompatible elements such as Li and K become enriched.

The highest concentration of Li (40 mg kg^‒1^) was found in interstitial brine of Bittern Pond (bit-C-I, Table 2). According to new given cut-off grade introduced by advanced industries, concentrations of Li in brine should be 25 mg kg^‒1^ or higher^49^ which are promising for lithium exploration and extraction purposes by advanced industries.

Other elements, especially Mg might control the Li concentration at most stages of evaporation/precipitation^41^. The main limitation of lithium extraction from brine is the difficulty in processing brine with a high magnesium to lithium ratio (Mg/Li)^50^. High Mg/Li ratios also complicate the extraction dynamics which could be attributed to the similar geochemical behavior of Mg^2+^ and Li^+^^51^. Separation of lithium and magnesium has been a major challenge due to their similar ionic properties^52^. This makes lithium extraction from brines with high Mg/Li ratios a difficult task, as additional steps and costs are required to reduce the magnesium content of the brine solution to an acceptable level^52^. Technically, a Mg/Li ratio not higher than 6 is required for conventional extraction of Li from brines by solar evaporation^41^. For Li recovery from brines, the removal of Mg is frequently necessary and the established method to this is selective magnesium precipitation using chemical reagents^53^.

The Mg/Li ratio in the Bam brines was 70 for sample bit-C-I, about 100 for sample t5, and between 130 to 140 for other samples (Table 3, Supplementary Fig. S6). There is no clear trend in the Mg/Li ratio during the brine evolution. The samples t5 and bit-C-I were collected at a very late stage of the brine evolution, when the ponds were almost completely solidified. Thus, massive precipitation of Mg might be the reason for low values, which corresponds to the maximum Li concentrations in these two liquid samples. The relatively constant Mg/Li ratio during the brine evolution is consistent with a steady increase of both elements through evaporation according to their similar geochemical behavior, they had precipitation behavior that was very similar.

**Table 3:** Mg/Li ratio during brine evolution in ponds.

Sample name	Brine evolution	Brine type	Mg/Li
Base pond			
bp	1	Surface	149.54
Pond 10			
t1	2	Surface	149.47
t2	3	Surface	140.01
t3	4	Surface	139.52
t3-I	4	Interstitial	140.91
t4	5	Surface	142.41
t4-I	5	Interstitial	145.13
t5	6	Surface	101.54
Bittern pond			
bit-A	1	Surface	151.82
bit-A-I	1	Interstitial	136.98
bit-B	2	Surface	140.96
bit-B-I	2	Interstitial	141.91
bit-C	3	Surface	144.39
bit-C-I	3	Interstitial	70.80

Thus, the high Mg/Li ratios of the Bam salt plug brines make them unsuitable for conventional evaporation/extraction techniques. However, many brines such as Uyuni brine do not show a suitable chemical composition to recovering Li by the use of classical techniques^48^, or 78% of the salt-lake brines in Western China have high Mg^2+^/Li^+^ ratios up to 500^54^. In spite of specified characteristics, these deposits are considered as future resources for lithium, although their exploration depends on the development of new methods.

The issue of reducing magnesium content of high Mg/Li brines using traditional methods incurs additional costs, which limits lithium recovery in low Mg/Li brines and prevents existing lithium resources from becoming sufficient^55^. Therefore, there is an urgent need to develop new methods that can extract Li from brine with a high Mg/Li ratio. Several methods have been developed to separate lithium from brines, including: calcined leaching^56^, adsorption^57^, precipitation^58^, nanofiltration^59^, electrolysis^60^ and solvent extraction^61^.

### Origin of lithium-rich brines

Lithium is widespread and relatively uniformly distributed in the Earth's crust, but can be concentrated in acidic igneous rocks and clayey sedimentary rocks. Li, a highly reactive cation with a relatively small ionic radius, can easily be replaced by cations with sufficiently similar atomic radii (Mg^2+^, Fe^2+^, Al^3+^, Ti^4+^) in geochemical processes^62^. Lithium is rich in late pegmatite minerals such as mica, few pyroxene, tourmaline, certain sedimentary aluminosilicates, and phosphates^62^. High Li contents were observed in rhyolitic rocks (samples 2, 7), and shale (sample 6) (Supplementary Table S2). Lithological observations showed that sample 6 consists of quartz, albite, calcite, clinochlore, and dickite.

The rhyolite samples are dominantly composed of mica, albite, microcline, and quartz; therefore, we suggest that Li was released from those samples^14^. Lithium is highly soluble and mobile during weathering, so it moves easily in arid climates along with other soluble salts such as, chlorides, sulfates, and borates^62^.

We found Li in markedly increased concentrations in clay-type samples (shale) and rhyolites which is in accordance with its strong chemical diagonal correlation with Al and Mg.

The geochemical behavior of Li in clay minerals differs from that of Na, K, Rb, Cs, Mg, and Ca because it strongly is bound during the formation of clay minerals, but is eventually released during clay decomposition. Nevertheless, Li is easily displaced by other cations and is a very mobile element that moves readily into and out of solutions and sediments^15^. Given the passage of brine-source streams through Li-bearing rhyolites and clays, we suggest that Li has originated from these rock types. In other words, lithium brine deposits result from the accumulation of lithium-rich brine in evaporation ponds. These brines typically result from weathering and leaching of lithium-bearing rocks (rhyolite volcanic rocks and shales), and over time they become concentrated by evaporation and precipitation. However, more chemical and isotopic analyzes are needed to accurately determine the origin of brine and lithium-bearing salts.

### Potential sources of lithium

There is no general agreement on the factors of Li enrichment in brines compared with the solid materials. Possible candidates are selective weathering of felsic volcanic rocks, geothermal activity related to volcanic systems, or underlying bodies of magma^63^. The rock-water interaction can also change the brine composition. Possible sources of Li in the Bam ponds may be (1) re-dissolution of Li from salt-bearing sediments; Li is easily leached from earlier salt sediments due to their weak resistance to weathering^64^. The salt shell has a relatively high Li content, and Li^+^ can replace Na^+^ in rock salt, but the relatively large difference in ionic radius severely limits this cation replacement^65^. Li is most likely trapped as brine inclusions within halite crystals rather than within other mineral structures^66^. (2) weathering and leaching from Li-rich volcanic rocks (rhyolite); lithium-bearing pegmatite minerals are formed by the crystallization of magma, and are typically found in granitic or metamorphic rocks. Due to the incompatibility of lithium during the crystallization process, pegmatite often concentrates lithium in the final stage of magma solidification, (3) release from sedimentary source rocks such as shale and marl; lithium content in shale, like other sedimentary rocks, generally depends on clastic sediment sources, changes during transport and post-depositional processes^67^. Li is mainly structurally bound to silicate minerals (such as clay and feldspar). During the continental weathering, Li preferentially incorporates into clays^68^. On the other hand, sediments with less clay, such as sand and silt, have a lower overall Li content.

The field and petrographic studies revealed penetrative alteration and weathering on the volcanic outcrops in this area. As it has been documented that rhyolites and shales can be highly enriched in Li and the results of the analysis of rock units in the region showed a higher amount of lithium in rhyolite rocks and shales, therefore, it can be concluded that these rock types have been subjected to leaching by meteoric waters in geological times. Li is mainly transported in solution to ponds and large amounts of Li-bearing solutions have been drained to the Bam salt plug basin. These preliminary results need further support by isotope-based geochemical studies.

A number of studies investigating lithium-bearing brines have confirmed the presence of rhyolite and shale units in the source, consistent with the possible source results of this study (e.g., Ryabtsev 2002; Munk 2011, 2013, 2014; Kesler, 2012; Phan 2016; Sarchi 2023).

## Conclusions

Preliminary petrographic and geochemical studies were conducted on the Bam salt plug, southern Iran, focusing on changes in Li concentration, composition of brine, evaporation products and assessing if the brines are potential sources for Li. The results are as follows:

1. The dominant rocks and minerals in the study area are shale, limestone, halite, gypsum, iron oxides, andesite, granodiorite, and rhyolite. Rhyolite is of high-K calc-alkaline and shoshonitic type, generated in an active continental margin.
2. According to the Piper diagram, the brine is of the sodium chloride type.
3. evaporative minerals precipitating from the borders to the center of the brine ponds are gypsum and halite as Li does not precipitate as an independent mineral phase. The distribution of Li in the precipitated sediments of the ponds decreased from the borders to the center so when precipitating, Li is concentrated in gypsum, while the deposited halite is almost depleted of Li.
4. The abundances of K, Li, Mg, and S increase in brine during evaporation, while Ca decreases. A general decrease of Na and Cl contents was observed following marked initial increases. Therefore, with the progressive evaporation and precipitation of gypsum and halite, the elements Ca and Na decrease in the brine and incompatible elements such as Li become enriched.
5. Li content of brine steadily decreases from the interstitial brine – surface brine to base pond, parallel to the brine evolution.
6. Lithium is present as dissolved Li^+^ in the brine and concentrates in halite during the brine evolution by a factor of up to 28 and with values up to 40 mg kg^‒1^.
7. Considering that 25 mg kg^‒1^ is a suitable concentration of Li for exploration purposes, brines from this salt plug can be suggested as an exploration potential source.
8. The Mg/Li ratios of more than 70 found in all evaporation ponds are too high for conventional evaporation/extraction techniques for which the Mg/Li ratio should not exceed 6. Thus, the Bam salt plug brines may only be exploited economically, if suitable techniques were developed to separate Li from the excess of Mg in another way than evaporative enrichment.
9. Bam salt plug in southern Iran is similar to other salt plugs due to the deposition of clays, evaporates and felsic igneous rocks that occur during the Sabkha and Playa periods. Li can be released from felsic rocks such as rhyolite, as well as from shale, evaporates, or salt sediments to form Li-enriched brines. Therefore, this study could be expanded to other salt plugs to assess their potential for Li exploration.
10. Further geochemical and isotopic investigations are needed to validate the proposed sources of lithium enrichment.

### Supplementary Information

Supplementary Information.

## Data Availability

All data generated or analysed during this study are included in this published article and the supplementary information file.

## References

[1] Manrique CJ, Martínez VE, Calderón ME, Guamán JG (2023). Geochemical characterization of lithium-bearing brines from Guaranda. Ecuador. Bol. Geol. (Univ. Ind. Santander).

[2] Liu Y, Zhang R, Wang J, Wang Y (2021). Current and future lithium-ion battery manufacturing. IScience.

[3] Jaskula, B. W. Lithium. Mineral commodity summaries. In *US Geological Survey*. 108–109 (2023).

[4] Guzman JI, Retamal C, Faúndez P, Joaquín Jara J (2022). Evolution of the surface area of critical lagoon systems in the Salar de Atacama. Nat. Res. Res..

[5] Tracy, B. S. Critical minerals in electric vehicle batterie. In *CRS Report R47227*. *Congressional Research Service*. https://crsreports.congress.gov/product/pdf/R/R47227 (2022).

[6] Yu X, Wang C, Huang H, Wang J, Yan K (2023). Lithium and brine geochemistry in the Qianjiang Formation of the Jianghan Basin, central China. Sci. Rep..

[7] Lusty, P. *et al.* Study on future UK demand and supply of lithium, nickel, cobalt, manganese and graphite for electric vehicle batteries. In *British Geological Survey Commissioned Report, CR/22/079* (2022).

[8] Dessemond C, Lajoie-Leroux F, Soucy G, Laroche N, Magnan JF (2019). Spodumene: The lithium market, resources and processes. Minerals.

[9] Haddok, E. Salt 'n power: A first look at the lithium flats of Bolivia (slide show) *Sci. Am.* (2010).

[10] Jaskula, B. W. Lithium. Mineral commodity summaries. In *US Geological Survey*. 98‒99 (2020).

[11] Marazuela MA, Ayora C, Vázquez-Suñé E, Olivella S, García-Gil A (2020). Hydrogeological constraints for the genesis of the extreme lithium enrichment in the Salar de Atacama (NE Chile): A thermohaline flow modelling approach. Sci. Total Environ..

[12] Ruiz-Leotaud, V. New project to investigate if California’s lithium valley is world’s largest brine source of lithium. In *Mining.com*. https://www.mining.com/new-project-to-investigate-if-californiaslithium-valley-is-the-worlds-largest-brine-source-of-lithium/ (2022).

[13] Jaskula, B. W. Lithium. Mineral commodity summaries. In *US Geological Survey*. 100‒101 (2022).

[14] Neukampf J, Ellis BS, Magna T, Laurent O, Bachmann O (2019). Partitioning and isotopic fractionation of lithium in mineral phases of hot, dry rhyolites: The case of the Mesa Falls Tuff, Yellowstone. Chem. Geol..

[15] Zhang JW (2021). Lithium and its isotopes behavior during incipient weathering of granite in the eastern Tibetan Plateau, China. Chem. Geol..

[16] Rankama K, Sahama TG (1955). Geochemistry.

[17] Gruber PW (2011). Global lithium availability: A constraint for electric vehicles?. J. Ind. Ecol..

[18] British Geological Survey. *Mineral Profile: Lithium*. https://www.bgs.ac.uk/downloads/start.cfm?id=3100. Accessed 08 Feb 2018 (2016).

[19] Mason B, Moore CB (1985). Principles of Geochemistry.

[20] Cannon, H. L., Harms, T. F. & Hamilton, J. C. Lithium in unconsolidated sediments and plants of the Basin and Range province, southern California and Nevada (No. 918). In *US Government Print. Office*. https://pubs.er.usgs.gov/publication/pp918 (1975).

[21] Li L (2018). Lithium recovery from aqueous resources and batteries: A brief review. Johnson Matthey Technol. Rev..

[22] Rosen MR (2020). Li and Ca enrichment in the bristol dry lake brine compared to brines from Cadiz and Danby dry lakes, Barstow-Bristol Trough, California, USA. Minerals.

[23] Sabins, F. F. International basement tectonics association publication. In *Proceedings of the Fourth International Conference on Basement Tectonics*. *Basement Tectonics Committee Inc.* Vol. 4. 307 (1981).

[24] Ghazban F, Al-Aasm IS (2010). Hydrocarbon-induced diagenetic dolomite and pyrite formation associated with the Hormoz Island salt dome, offshore Iran. J. Pet. Geol..

[25] Ahmadzadeh Heravi, M., Houshmandzadeh, A. & Nabavi, M. H. New concept of Hormuz formation’s stratigraphy and the problem of salt diapirism in south Iran. In *Proceeding Symposium on Diapirism with Special Reference to Iran*. *Geological Survey of Iran, Tehran*. 1‒22 (1990).

[26] Garcia MG, Borda LG, Godfrey LV, Steinmetz RL, Losada-Calderon A (2020). Characterization of lithium cycling in the Salar De Olaroz, Central Andes, using a geochemical and isotopic approach. Chem. Geol..

[27] David, M., Lyth, S. M., Lindner, R. & Harrington, G.F. Critical raw materials. In *Future-Proofing Fuel Cells*. (Palgrave Macmillan, 2021).

[28] Shams R, Fard IA, Bouzari S, Pourkermani M (2020). Investigating role of the Hormuz salt bodies in initiation and evolution of the strike slip faults in the Fars Zone of the Zagros fold and thrust belt: Insights from seismic data and sandbox modeling. Pure Appl. Geophys..

[29] Sarkarinejad K, Faghih A, Grasemann B (2008). Transpressional deformations within the Sanandaj-Sirjan metamorphic belt (Zagros Mountains, Iran). J. Struct. Geol..

[30] Rashidi A (2020). Morphotectonic and earthquake data analysis of interactional faults in Sabzevaran Area, SE Iran. J. Struct. Geol..

[31] Ghanbarian MA, Yassaghi A, Derakhshani R (2021). Detecting a sinistral transpressional deformation belt in the Zagros. Geosciences.

[32] Smith AG (2012). A review of the Ediacaran to Early Cambrian (‘Infra-Cambrian’) evaporites and associated sediments of the Middle East. Geol. Soc. Spec. Publ..

[33] Thomas RJ, Ellison RA, Goodenough KM, Roberts NM, Allen PA (2015). Salt domes of the UAE and Oman: Probing eastern Arabia. Precambrian Res..

[34] Jahani S, Callot JP, Letouzey J, Frizon de Lamotte D (2009). The eastern termination of the Zagros Fold-and-Thrust Belt, Iran: Structures, evolution, and relationships between salt plugs, folding, and faulting. Tectonics.

[35] Motamedi H, Sepehr M, Sherkati S, Pourkermani M (2011). Multi-phase Hormuz salt diapirism in the southern Zagros, SW Iran. J. Pet. Geol..

[36] Asadpour G (2015). Evaluating the geochemistry of Bam salt dome in Hormozgan Province, Iran. Pollution.

[37] Esri, R. *ArcGIS Desktop: Release 10*. (Environmental System Research Institute, 2011).

[38] Bosák P, Jaroš J, Spudil J, Sulovský P, Vaclavek V (1998). Salt plugs in the Eastern Zagros, Iran: Results of regional geological reconnaissance. GeoLines.

[39] Risacher F, Fritz B (1991). Geochemistry of Bolivian salars, Lipez, southern Altiplano: Origin of solutes and brine evolution. Geochim. Cosmochim. Acta.

[40] Robb L (2013). Introduction to Ore-Forming Processes.

[41] Liu X, Zhong M, Chen X, Zhao Z (2018). Separating lithium and magnesium in brine by aluminum-based materials. Hydrometallurgy.

[42] Singh AK, Kumar SR (2015). Quality assessment of groundwater for drinking and irrigation use in semi-urban area of Tripura, India. Eco. Environ. Conserv..

[43] Hastie AR, Kerr AC, Pearce JA, Mitchell SF (2007). Classification of altered volcanic island arc rocks using immobile trace elements: Development of the Th–Co discrimination diagram. J. Petrol..

[44] Pearce JA (1982). Trace element characteristics of lavas from destructive plate boundaries. Andesites Orog. Andesites Relat. Rocks.

[45] Pearce JA, Harris NB, Tindle AG (1984). Trace element discrimination diagrams for the tectonic interpretation of granitic rocks. J. Petrol..

[46] Schandl ES, Gorton MP (2002). Application of high field strength elements to discriminate tectonic settings in VMS environments. Econ. Geol..

[47] Rettig SL, Jones BF, Risacher F (1980). Geochemical evolution of brines in the Salar of Uyuni. Bolivia. Chem. Geol..

[48] Ogawa Y, Koibuchi H, Suto K, Inoue C (2014). Effects of the chemical compositions of S alars de U yuni and A tacama Brines on lithium concentration during evaporation. Resour. Geol..

[49] Song W, Gang H, Ma Y, Yang S, Mu B (2017). Migration behavior of lithium during brine evaporation and KCl production plants in Qarhan salt lake. Minerals.

[50] Liu G, Zhao Z, He L (2020). Highly selective lithium recovery from high Mg/Li ratio brines. Desalination.

[51] Guo X, Hu S, Wang C, Duan H, Xiang X (2018). Highly efficient separation of magnesium and lithium and high-valued utilization of magnesium from salt lake brine by a reaction-coupled separation technology. Ind. Eng. Chem. Res..

[52] Shi W (2019). Efficient lithium extraction by membrane capacitive deionization incorporated with monovalent selective cation exchange membrane. Sep. Purif. Technol..

[53] An JW, Kang DJ, Tran KT, Lim T, Tran T (2012). Recovery of lithium from Uyuni salar brine. Hydrometallurgy.

[54] Song JF, Nghiem LD, Li XM, He T (2017). Lithium extraction from Chinese salt-lake brines: Opportunities, challenges, and future outlook. Environ. Sci. Water Res. Technol..

[55] Bai R, Wang J, Wang D, Cui J, Zhang Y (2022). Recovery of lithium from high Mg/Li ratio salt-lake brines using ion-exchange with NaNTf2 and TBP. Hydrometallurgy.

[56] Meshram P, Pandey BD, Mankhand TR (2014). Extraction of lithium from primary and secondary sources by pre-treatment, leaching and separation: A comprehensive review. Hydrometallurgy.

[57] Yang G (2020). Capturing lithium using functional macroporous microspheres with multiple chambers from one-step double emulsion via a tailoring supramolecular route and postsynthetic interface modification. Chem. Eng. J..

[58] Ooi K (2017). Recovery of lithium from salt-brine eluates by direct crystallization as lithium sulfate. Hydrometallurgy.

[59] Wu H (2020). A novel nanofiltration membrane with [MimAP][Tf2N] ionic liquid for utilization of lithium from brines with high Mg^2+^/Li^+^ ratio. J. Membr. Sci..

[60] Ji L (2016). Lithium extraction with a synergistic system of dioctyl phthalate and tributyl phosphate in kerosene and FeCl_3_. Hydrometallurgy.

[61] Bai R, Wang J, Wang D, Zhang Y, Cui J (2021). Selective separation of lithium from the high magnesium brine by the extraction system containing phosphate-based ionic liquids. Sep. Purif. Technol..

[62] Kabata-Pendias A, Mukherjee AB (2007). Trace Elements from Soil to Human.

[63] Munk LA, Boutt DF, Hynek SA, Moran BJ (2018). Hydrogeochemical fluxes and processes contributing to the formation of lithium-enriched brines in a hyper-arid continental basin. Chem. Geol..

[64] Risacher F, Fritz B (2009). Origin of salts and brine evolution of Bolivian and Chilean salars. Aquat. Geochem..

[65] Sarchi C (2023). Lithium enrichment in the Salar de Diablillos, Argentina, and the influence of Cenozoic volcanism in a basin dominated by Paleozoic basement. Miner. Depos..

[66] Godfrey LV (2013). The role of climate in the accumulation of lithium-rich brine in the Central Andes. Appl. Geochem..

[67] Phan T (2016). Factors controlling Li concentration and isotopic composition in formation waters and host rocks of Marcellus Shale, Appalachian Basin. Chem. Geol..

[68] Liu XM, Wanner C, Rudnick RL, McDonough WF (2015). Processes controlling δ7Li in rivers illuminated by study of streams and groundwaters draining basalts. Earth Planet. Sci. Lett..

