# Supplementary material for: Investigating the geochemical behavior and exploration potential of lithium in brines; a case study of Bam salt plug, Zagros Zone, southern Iran
Source: Sci Rep. 2023 Dec 7;13:21567. doi: 10.1038/s41598-023-48909-5 (PMC10700501; doi:10.1038/s41598-023-48909-5)
Supplement: Supplementary file 1 — Supplementary Information. [file 41598_2023_48909_MOESM1_ESM.docx]

**Supplementary Information**

**for**

**Investigating the geochemical behavior and exploration potential of lithium in brines; a case study of Bam salt plug, Zagros Zone, southern Iran**

Marziyeh Bazamad^1,^ Majid H. Tangestani^1,^*, Sina Asadi^1^ and Michael Staubwasser^2^

^1^ Department of Earth Sciences, Faculty of Sciences, Shiraz University, Shiraz, 71454 Iran (I.R.).

Prof. Dr. Majid H. Tangestani, Email: [tangstan@shirazu.ac.ir](mailto:tangstan@shirazu.ac.ir), ORCID: 0000-0003-2503-9940 (MT).

Marziyeh Bazamad, Email: [mbazamad@shirazu.ac.ir](mailto:mbazamad@shirazu.ac.ir), ORCID: 0000-0003-2289-6250 (MB).

Assoc.-Prof. Dr. Sina Asadi, Email: [sinaasadi@shirazu.ac.ir](mailto:sinaasadi@shirazu.ac.ir), ORCID: [0000-0003-3074-1734](https://orcid.org/0000-0003-3074-1734) (SA).

^2^ Universität zu Köln, Mathematisch-Naturwissenschaftliche Fakultät, Department für Geowissenschaften, Institut für Geologie und Mineralogie.

Prof. Dr. Michael Staubwasser, Email: [m.staubwasser@uni-koeln.de](mailto:m.staubwasser@uni-koeln.de), ORCID: 0000-0002-5892-1115 (MS).

* Corresponding author:

Prof. Dr. Majid H. Tangestani, Email: [tangstan@shirazu.ac.ir](mailto:tangstan@shirazu.ac.ir), Tel.: +98 9173170200

Contents:

**Figure S1.** Dominant sedimentary units in the Bam salt plug; **(a)** marl, **(b)** limestone, gypsum and halite, **(c)** halite, **(d)** carbonates, marl, shale, siltstone, halite, and sandstone, **(e)** shale, and **(f)** grayish red, grayish, and purple red shale, marl, sandstone, and carbonates.

**Figure S2.** The dominant magmatic rocks units in the Bam salt plug; **(a)** gabbro, **(b)** andesite, **(c)** rhyolite, and **(d)** granodiorite.

**Figure S3.** Powder XRD plot of the Bittern pond sediment which calculated stick plots of halite (01-075-0306) and gypsum (00-003-0053).

**Figure S4.** Trace element correlations in Bam rhyolites plotted as log mg kg^‒1^ amounts. **(a)** Co vs. Th (Hastie et al., 2007), and **(b)** Ta/Yb vs. Th/Yb (Pearce, 1982) diagrams are shoshonite rhyolites, and based on the diagrams of **(c)** Rb vs. Y+Nb, **(d)** Ta vs. Yb, **(e)** Rb vs. Y+Nb, and **(f)** Th/Yb vs. Nb/Yb are plotted in the “volcanic arc” domain (VAG).

**Figure S5.** Geotectonic classification of volcanic rocks of the Bam salt plug based on correlation plots. **(a)** Th/Yb vs. Ta/Yb diagram, **(b)** Th vs. Ta diagram, **(c)** Th/Hf vs. Ta/Hf diagram, and **(d)** Th/Ta vs. Yb diagram (Schandl and Gorton, 2002).

**Figure S6.** Mg/Li ratio against brine evolution a: in the ponds brine b: in the Bittern brine.

**Table S1.** Operating parameters for ICP-OES analyses.

**Table S2.** Major (Wt%) and trace (mg kg^‒1^) element contents of solid samples in the Bam salt plug.

**Table S3.** Boron-normalized value (NB) changes in the ponds of the Bam salt plug.


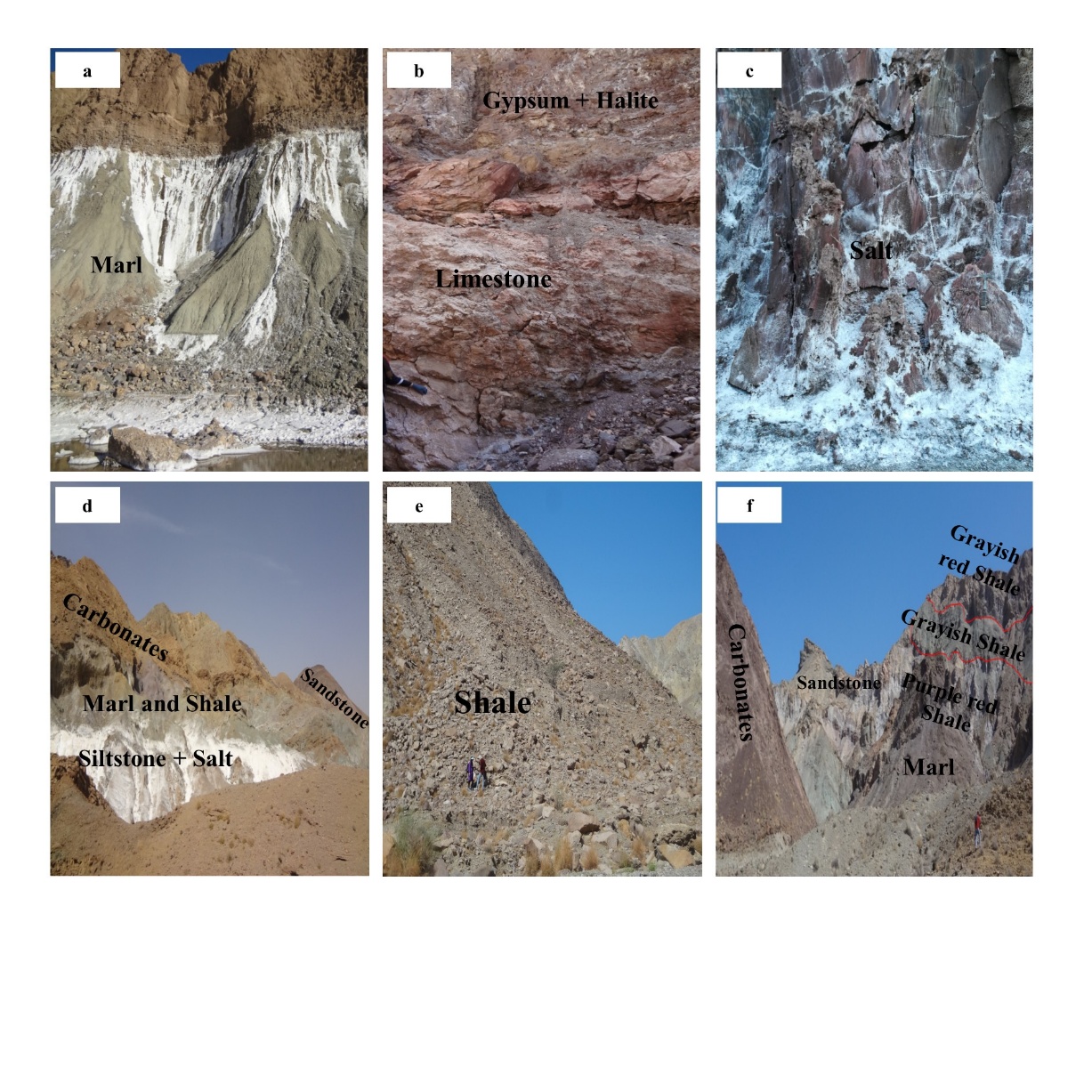


**Figure S1.** Dominant sedimentary units in the Bam salt plug; **(a)** marl, **(b)** limestone, gypsum and halite, **(c)** halite, **(d)** carbonates, marl, shale, siltstone, halite, and sandstone, **(e)** shale, and **(f)** grayish red, grayish, and purple red shale, marl, sandstone, and carbonates.

**
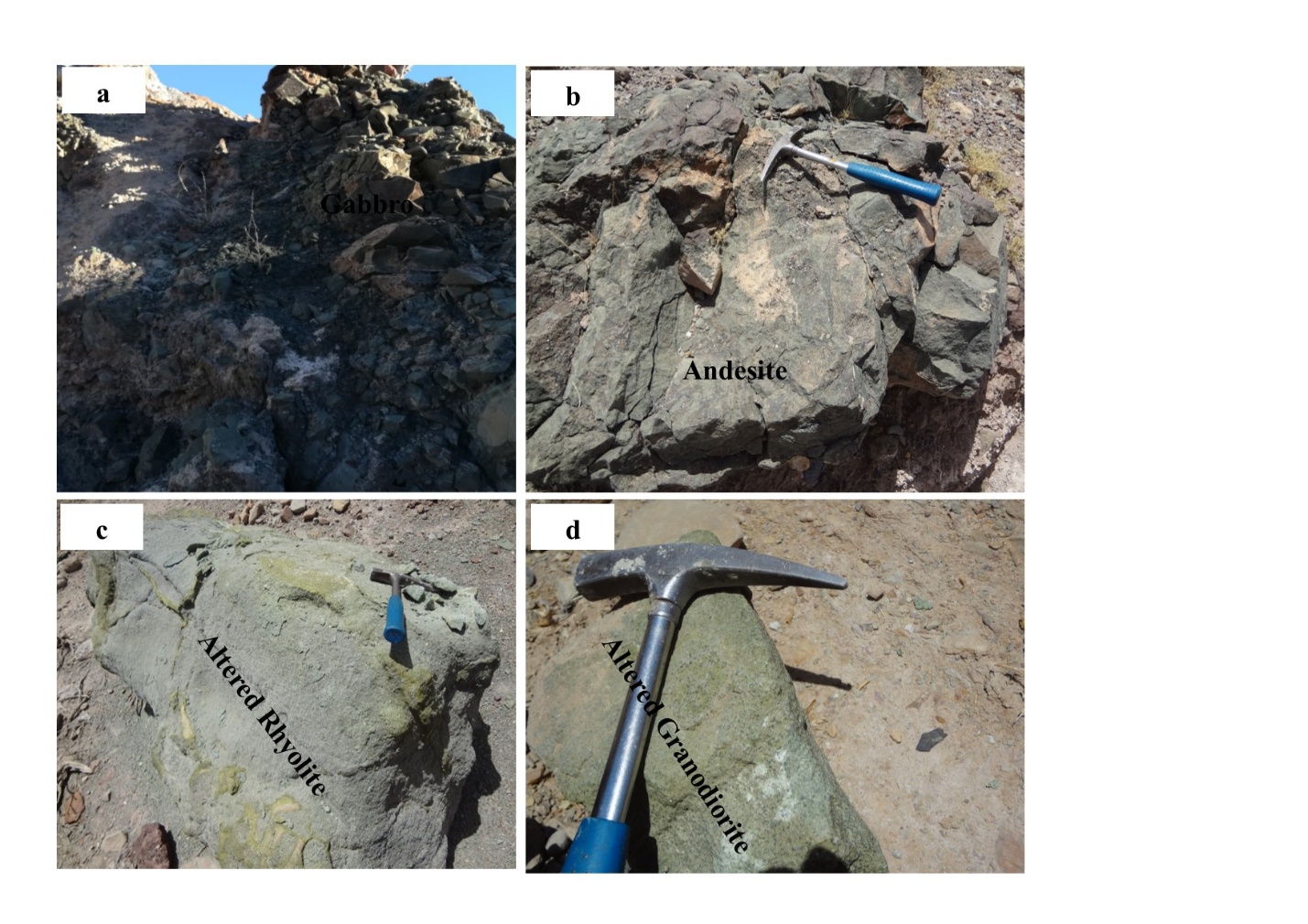
**

**Figure S2.** The dominant magmatic rocks units in the Bam salt plug; **(a)** gabbro, **(b)** andesite, **(c)** rhyolite, and **(d)** granodiorite.


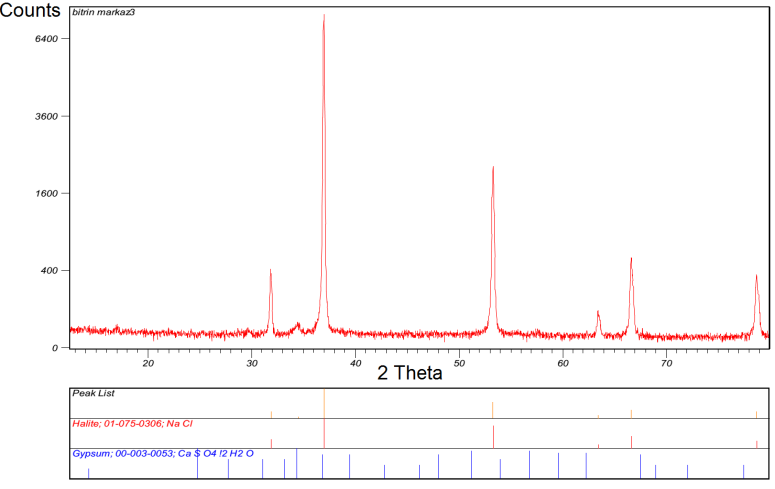


**Figure S3.** Powder XRD plot of the Bittern pond sediment which calculated stick plots of halite (01-075-0306) and gypsum (00-003-0053).


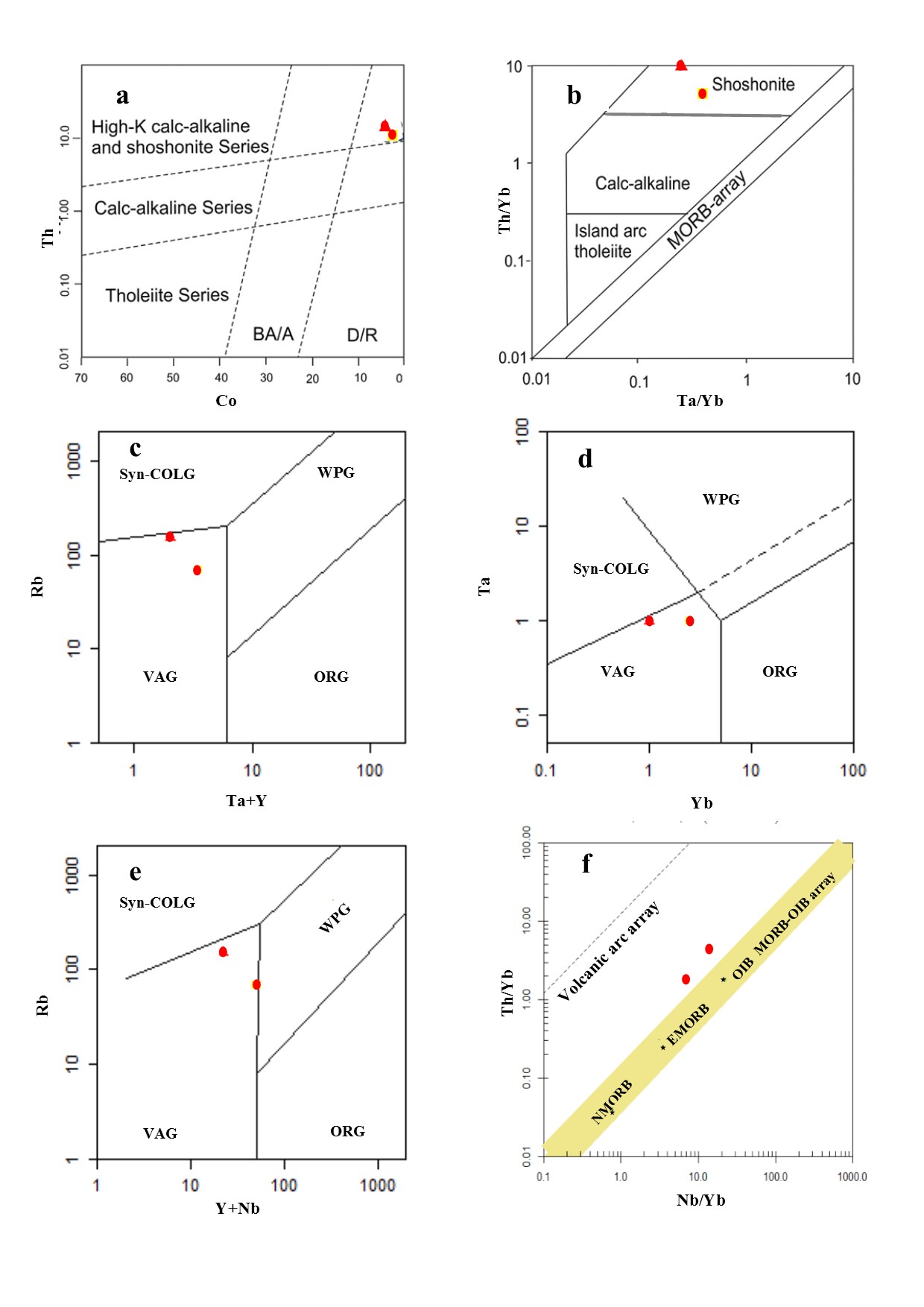


**Figure S4.** Trace element correlations in Bam rhyolites plotted as log mg kg^‒1^ amounts. **(a)** Co vs. Th (Hastie et al., 2007), and **(b)** Ta/Yb vs. Th/Yb (Pearce, 1982) diagrams are shoshonite rhyolites, and based on the diagrams of **(c)** Rb vs. Y+Nb, **(d)** Ta vs. Yb, **(e)** Rb vs. Y+Nb, and **(f)** Th/Yb vs. Nb/Yb are plotted in the “volcanic arc” domain (VAG).


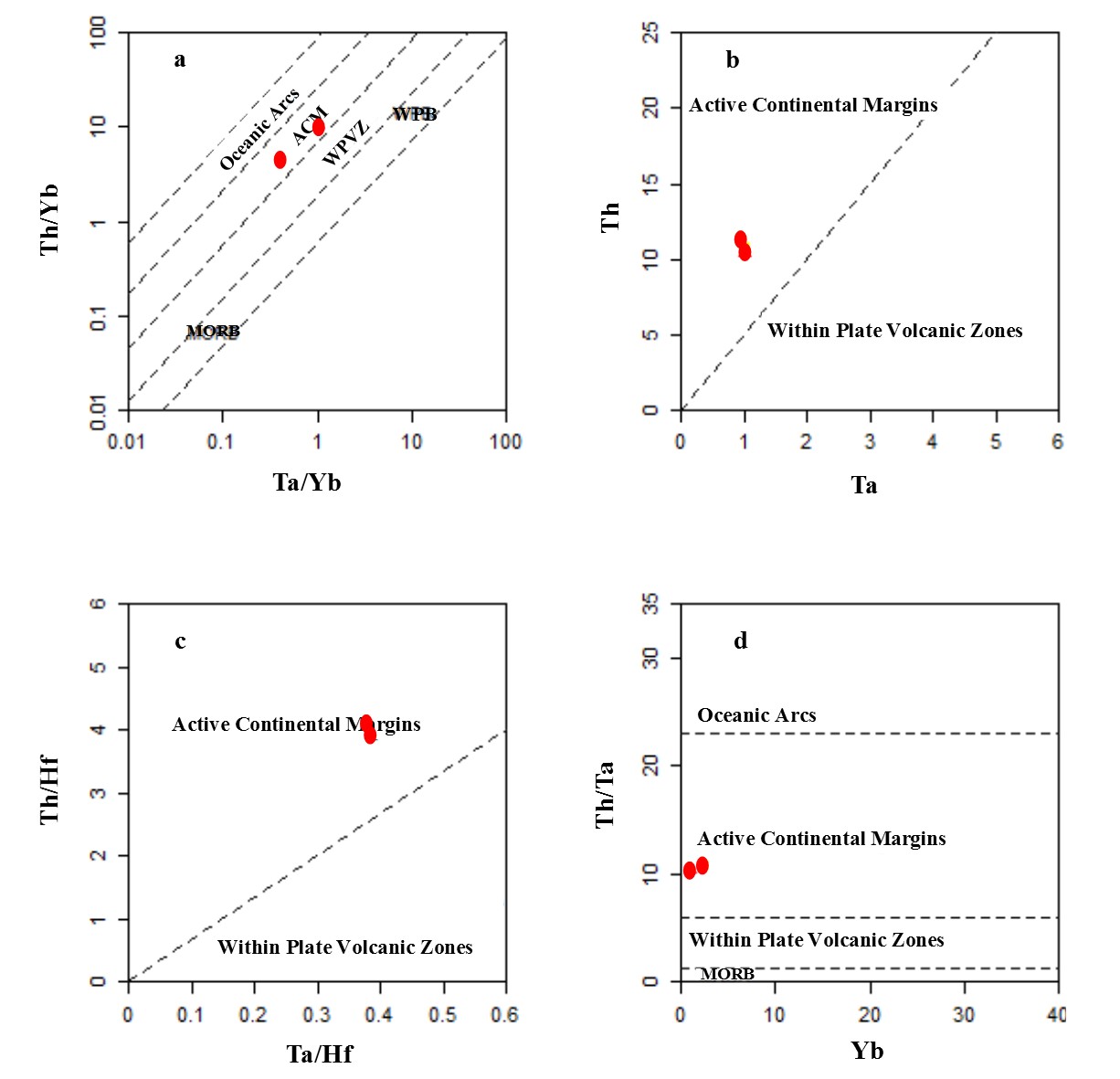


**Figure S5.** Geotectonic classification of volcanic rocks of the Bam salt plug based on correlation plots. **(a)** Th/Yb vs. Ta/Yb diagram, **(b)** Th vs. Ta diagram, **(c)** Th/Hf vs. Ta/Hf diagram, and **(d)** Th/Ta vs. Yb diagram (Schandl and Gorton, 2002).


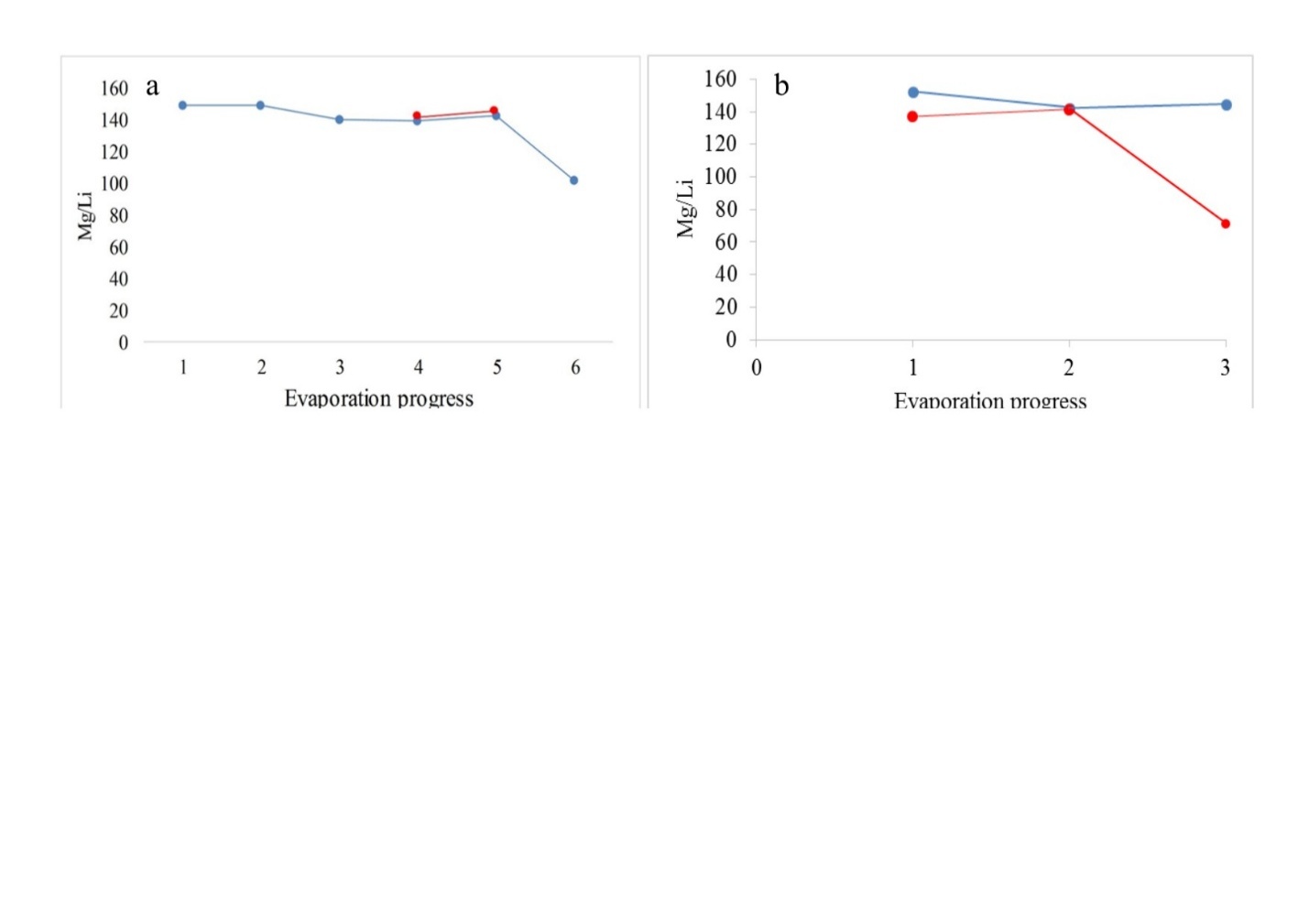


**Figure S6.** Mg/Li ratio against brine evolution **(a)** in the ponds brine **(b)** in the Bittern brine.

Blue color: surface brines, Red color: interstitial brines.

| **Parameter Setting** | |
| --- | --- |
| RF power | 1400 W |
| Pump speed | 30 rpm |
| Auxiliary gas flow | 1.3 l·min-1 |
| Coolant gas flow | 14.5 l·min-1 |
| Nebulizer gas flow | 0.7 l·min-1 |
| Nebulizer | Cross-flow |
| Spray chamber | Scott double pass |
| Torch | Quartz for axial view |
| Sample uptake | 0.2 ml·min-1 |

**Table S1.** Operating parameters for ICP-OES analyses.

| sample | **1** | **2** | **3** | **4** | **5** | **6** | **7** |  | **Bit-1^b^** | **Bit-2** | **Bit-3** | **Bit-4** | **12-1^c^** | **12-2** | **12-3** | **12-4** | **10-1^d^** | **10-2** | **10-3** | **10-4** |
| --- | --- | --- | --- | --- | --- | --- | --- | --- | --- | --- | --- | --- | --- | --- | --- | --- | --- | --- | --- | --- |
| latitude | 27°14'02" | 27°14'54" | 27°14'24" | 27°13'46" | 27°15'09" | 27°14'17" | 27°16'25" |  | 27°12'45" | 27°12'45" | 27°12'45" | 27°12'45" | 27°13'30" | 27°13'30" | 27°13'30" | 27°13'30" | 27°12'47.30" | 27°12'48" | 27°12'48.30" | 27°12'49" |
| longitude | 54°52'42" | 54°52'48" | 54°53'02" | 54°52'40" | 54°52'58" | 54°52'52" | 54°54'57" |  | 54°52'14.30" | 54°52'15" | 54°52'15.30" | 54°52'16" | 54°52'07" | 54°52'08" | 54°52'09" | 54°52'10" | 54°52'14" | 54°52'14" | 54°52'14" | 54°52'14" |
|  | andesite | rhyolite | diorite | gypsum | tuff | shale | rhyolite |  | salt | salt | salt | salt + gypsum | salt | salt | salt | salt + gypsum | salt | salt | salt | salt + gypsum |
| major elements | | |  |  |  |  |  |  |  |  |  |  |  |  |  |  |  |  |  |  |
| Ca | 3.35 | 0.13 | 4.03 | 3.75 | 6.08 | 1.65 | 1.1 |  | 0.82 | 0.43 | 0.73 | 0.52 | 0.18 | 0.39 | 0.53 | 0.4 | 0.52 | 0.53 | 0.48 | 0.51 |
| K | 2.1 | 1.9 | 2.1 | 0.1 | 0.2 | 1.6 | 1.4 |  | 0.1 | 0.2 | 0.3 | 0.3 | 0.1 | 0.2 | 0.2 | 0.7 | 0.1 | 0.2 | 0.2 | 0.2 |
| Li | 26 | 31 | 9.9 | 2 | <1 | 33 | 21 |  | <1 | 4.6 | 7 | 9.5 | <1 | <1 | 2.5 | 30 | 1.2 | 2.2 | 2.1 | 5.1 |
| Mg | 2.36 | 0.69 | 2.09 | 0.1 | 0.2 | 1.36 | 0.1 |  | <0.1 | 0.11 | 0.16 | 0.19 | <0.1 | 0.13 | 0.1 | 0.5 | <0.1 | <0.1 | <0.1 | <0.1 |
| Na | 0.3 | 1 | 0.3 | 0.2 | 0.1 | 1.2 | 1.7 |  | 5.3 | 5.1 | 5.2 | 5.1 | 5.3 | 5.2 | 5 | 4.9 | 5.1 | 5 | 4.8 | 4.7 |
| trace elements | | |  |  |  |  |  |  |  |  |  |  |  |  |  |  |  |  |  |  |
| Ag | <1 | 1.49 | <1 | <1 | <1 | <1 | <1 |  | <1 | <1 | <1 | <1 | <1 | <1 | <1 | <1 | <1 | <1 | <1 | <1 |
| Al | 5.59 | 2.31 | 5.62 | <0.1 | 0.25 | 4.18 | 3.35 |  | <0.1 | <0.1 | <0.1 | <0.1 | <0.1 | 0.22 | <0.1 | <0.1 | <0.1 | <0.1 | <0.1 | <0.1 |
| As | 17.3 | 11.8 | 8.57 | 8.2 | 6.91 | 22.9 | 12.1 |  | 8.32 | 8.81 | 9.53 | 7.23 | 8.99 | 9.26 | 8.2 | 9.46 | 8.32 | 9.04 | 7.63 | 9.5 |
| Ba | 1335 | 235 | 198 | 31.2 | 26.7 | 545 | 368 |  | 364 | 77.9 | 113 | 71.9 | 63.1 | 78.9 | 36.5 | 36.8 | 24 | 18.9 | 23.7 | 17.8 |
| Be | 0.17 | 1.46 | 0.65 | 0.1 | 0.1 | 1.23 | 5.21 |  | <0.1 | <0.1 | <0.1 | <0.1 | <0.1 | <0.1 | <0.1 | <0.1 | <0.1 | <0.1 | <0.1 | <0.1 |
| Bi | <1 | <1 | <1 | <1 | <1 | <1 | <1 |  | <1 | <1 | <1 | <1 | <1 | <1 | <1 | <1 | <1 | <1 | <1 | <1 |
| Cd | 0.33 | <0.1 | <0.1 | 0.13 | 0.49 | 0.56 | <0.1 |  | 2.63 | 0.1 | 0.26 | <0.1 | <0.1 | 0.16 | 0.45 | 0.18 | <0.1 | <0.1 | <0.1 | <0.1 |
| Ce | 5.48 | 35.9 | 22.6 | 7.23 | 12 | 31.4 | 170 |  | <1 | <1 | <1 | <1 | <1 | <1 | <1 | <1 | <1 | <1 | <1 | <1 |
| Co | 43 | 9.02 | 31.4 | <1 | <1 | 30 | 4.12 |  | <1 | <1 | <1 | <1 | <1 | 1.39 | <1 | <1 | <1 | <1 | <1 | <1 |
| Cr | 271 | 89.3 | 168 | 7.52 | 13.2 | 79.5 | 83.7 |  | 13.1 | 11.8 | 8.03 | 7.93 | 7.19 | 21.4 | 10 | 10.3 | 15.1 | 3.44 | 5.03 | 5.47 |
| Cs | 7.2 | 7.6 | 4.4 | 4.1 | 4.5 | 6.6 | 2.9 |  | 6.7 | 7.6 | 6 | 6.7 | 5.9 | 5.8 | 5.5 | 9.3 | 4.5 | 5.7 | 3.8 | 6.1 |
| Cu | 5.72 | 4.86 | 6.57 | 10.6 | 2.55 | 249 | 10.7 |  | 8.13 | 4.83 | 4.33 | 2.55 | 2.92 | 3.1 | 2.87 | 2.6 | 3.26 | 1.02 | 2.96 | 1.08 |
| Dy | 2.07 | 1.68 | 4.24 | <1 | <1 | 1.7 | 11.9 |  | <1 | <1 | <1 | <1 | <1 | <1 | <1 | <1 | <1 | <1 | <1 | <1 |
| Er | 1.09 | <1 | 2.11 | <1 | <1 | <1 | 5.48 |  | <1 | <1 | <1 | <1 | <1 | <1 | <1 | <1 | <1 | <1 | <1 | <1 |
| Eu | <1 | <1 | 1.31 | <1 | <1 | <1 | 4.58 |  | <1 | <1 | <1 | <1 | <1 | <1 | <1 | <1 | <1 | <1 | <1 | <1 |
| Fe | 4.06 | 0.68 | 5.67 | 0.1 | 0.18 | 2.06 | 3.41 |  | <0.1 | <0.1 | <0.1 | <0.1 | <0.1 | 0.18 | <0.1 | <0.1 | <0.1 | <0.1 | <0.1 | <0.1 |
| Ga | 169 | 36.6 | 36.9 | 3.34 | 2.61 | 65 | 63.1 |  | 25.3 | 3.78 | 4.68 | 3.21 | 4.21 | 8.13 | 2.91 | 3.14 | 1.81 | 2.11 | 1.64 | 2.51 |
| Gd | 2.52 | 2.75 | 5.03 | <1 | <1 | 2.06 | 19.9 |  | <1 | <1 | <1 | <1 | <1 | <1 | <1 | <1 | <1 | <1 | <1 | <1 |
| Hf | 1.05 | 1.53 | 1.51 | <1 | <1 | <1 | 2.67 |  | 1.13 | <1 | <1 | <1 | <1 | <1 | <1 | <1 | <1 | 1.37 | <1 | <1 |
| Hg | <1 | <1 | <1 | <1 | <1 | <1 | <1 |  | <1 | <1 | <1 | <1 | <1 | <1 | <1 | <1 | <1 | <1 | <1 | <1 |
| Ho | <1 | <1 | <1 | <1 | <1 | <1 | 2.08 |  | <1 | <1 | <1 | <1 | <1 | <1 | <1 | <1 | <1 | <1 | <1 | <1 |
| In | <1 | <1 | <1 | <1 | <1 | <1 | <1 |  | <1 | <1 | <1 | <1 | <1 | <1 | <1 | <1 | <1 | <1 | <1 | <1 |
| La | 1 | 9.46 | 7.51 | <1 | 1.05 | 8.37 | 50.9 |  | <1 | <1 | <1 | <1 | <1 | <1 | <1 | <1 | <1 | <1 | <1 | <1 |
| Lu | <1 | <1 | <1 | <1 | <1 | <1 | <1 |  | <1 | <1 | <1 | <1 | <1 | <1 | <1 | <1 | <1 | <1 | <1 | <1 |
| Mn | 1367 | 69 | 1080 | 20.5 | 103 | 703 | 2159 |  | 7.15 | 7.93 | 7.39 | 8.95 | 6.93 | 57.5 | 6.7 | 16.9 | 12.2 | 5.2 | 4.98 | 4.16 |
| Mo | <1 | <1 | <1 | <1 | <1 | 11.8 | 1.28 |  | <1 | <1 | <1 | <1 | <1 | <1 | <1 | 1.37 | <1 | <1 | <1 | <1 |
| Nb | 1.45 | 20.5 | 6.71 | <1 | <1 | 4.65 | 34.5 |  | <1 | <1 | <1 | <1 | <1 | <1 | <1 | <1 | <1 | <1 | <1 | <1 |
| Nd | 3.36 | 16.9 | 16 | <1 | 1.6 | 13.7 | 121 |  | <1 | <1 | <1 | <1 | <1 | <1 | <1 | <1 | <1 | <1 | <1 | <1 |
| Ni | 85.6 | 16.4 | 20.9 | 1.51 | 1.94 | 17.6 | 2.52 |  | 1.79 | 1.82 | 1.35 | 1.15 | 1.08 | 4.42 | 1.03 | 1.94 | 3.39 | 1.12 | <1 | 2.55 |
| P | 0.03 | 0.01 | 0.05 | 0.01 | 0.01 | 0.05 | 0.01 |  | <0.01 | 0.01 | <0.01 | <0.01 | 0.01 | 0.01 | <0.01 | <0.01 | <0.01 | <0.01 | <0.01 | <0.01 |
| Pb | 4.35 | 4.27 | 13.2 | 1.68 | 8.62 | 102 | 7.78 |  | 186 | 27.7 | 29.8 | 22.4 | 20.7 | 26.6 | 19.1 | 10.5 | 9.21 | 4.49 | 8.06 | 6.89 |
| Pr | 2.71 | 15.1 | 13.6 | <1 | 1.31 | 12.9 | 108 |  | <1 | <1 | <1 | <1 | <1 | <1 | <1 | <1 | <1 | <1 | <1 | <1 |
| Rb | 191 | 152 | 61 | 15 | 17 | 92 | 69 |  | 12 | 18 | 15 | 17 | 18 | 24 | 16 | 35 | 14 | 11 | 9.2 | 16 |
| S | 0.26 | 0.11 | <0.1 | 7.86 | 0.1 | 0.54 | 0.1 |  | 0.52 | 0.45 | 0.58 | 0.71 | 0.17 | 0.29 | 0.62 | 0.95 | 0.55 | 0.65 | 0.58 | 0.65 |
| Sb | 3.41 | 1.23 | <1 | <1 | <1 | 1.06 | <1 |  | <1 | 1.99 | 1.23 | <1 | <1 | <1 | <1 | <1 | <1 | <1 | <1 | <1 |
| Sc | 15.2 | <1 | 29.5 | <1 | <1 | 3.96 | <1 |  | <1 | <1 | <1 | <1 | <1 | <1 | <1 | <1 | <1 | <1 | <1 | <1 |
| Sm | 1.52 | 2.26 | 3.17 | <1 | <1 | 1.98 | 23 |  | <1 | <1 | <1 | <1 | <1 | <1 | <1 | <1 | <1 | <1 | <1 | <1 |
| Sn | 1.17 | 5.42 | 1.17 | <1 | <1 | 1.27 | 7.76 |  | <1 | <1 | <1 | <1 | <1 | <1 | <1 | <1 | 1.01 | <1 | <1 | <1 |
| Sr | 283 | 11.6 | 140 | 788 | 152 | 98.6 | 50.8 |  | 85.9 | 81.1 | 107 | 111 | 22.4 | 38.3 | 186 | 123 | 70.1 | 130 | 155 | 175 |
| Ta | <1 | <1 | <1 | <1 | <1 | <1 | <1 |  | <1 | <1 | <1 | <1 | <1 | <1 | <1 | <1 | <1 | <1 | <1 | <1 |
| Tb | <1 | <1 | <1 | <1 | <1 | <1 | 2.17 |  | <1 | <1 | <1 | <1 | <1 | <1 | <1 | <1 | <1 | <1 | <1 | <1 |
| Te | 4.07 | 14.9 | 1.36 | 1.36 | 4.75 | <1 | <1 |  | 5.43 | 6.11 | 8.15 | 4.07 | 10.9 | 10.9 | 8.83 | 9.51 | <1 | <1 | 1.36 | 1.36 |
| Th | <1 | 10.5 | 1.76 | <1 | <1 | 4.5 | 10.8 |  | <1 | <1 | <1 | <1 | <1 | <1 | <1 | <1 | <1 | <1 | <1 | <1 |
| Ti | 0.3 | 0.08 | 0.46 | 0.01 | 0.01 | 0.12 | 0.12 |  | <0.01 | <0.01 | <0.01 | <0.01 | <0.01 | 0.01 | <0.01 | <0.01 | <0.01 | <0.01 | <0.01 | <0.01 |
| Tl | <1 | <1 | <1 | <1 | <1 | <1 | <1 |  | <1 | <1 | <1 | <1 | <1 | <1 | <1 | <1 | <1 | <1 | <1 | <1 |
| Tm | <1 | <1 | <1 | <1 | <1 | <1 | <1 |  | <1 | <1 | <1 | <1 | <1 | <1 | <1 | <1 | <1 | <1 | <1 | <1 |
| U | <1 | 1.86 | <1 | <1 | 1.25 | 1.68 | <1 |  | <1 | <1 | <1 | <1 | <1 | <1 | <1 | <1 | <1 | <1 | <1 | <1 |
| V | 119 | 10.7 | 154 | <1 | 7.52 | 38.8 | 4.36 |  | <1 | <1 | <1 | <1 | <1 | 3.42 | <1 | 1.01 | <1 | <1 | <1 | <1 |
| W | <1 | 1.11 | <1 | <1 | <1 | <1 | <1 |  | <1 | <1 | <1 | <1 | <1 | <1 | <1 | <1 | <1 | <1 | <1 | <1 |
| Y | 3.37 | 1.97 | 7.49 | <1 | <1 | 2.29 | 15.4 |  | <1 | <1 | <1 | <1 | <1 | <1 | <1 | <1 | <1 | <1 | <1 | <1 |
| Yb | <1 | <1 | <1 | <1 | <1 | <1 | 2.43 |  | <1 | <1 | <1 | <1 | <1 | <1 | <1 | <1 | <1 | <1 | <1 | <1 |
| Zn | 57 | 18.8 | 64.6 | 11.2 | 40.7 | 69.5 | 36.9 |  | 49 | 22.3 | 29.5 | 26 | 9.07 | 13.5 | 41.2 | 49.1 | 8.54 | 13.5 | 15.1 | 16.7 |
| Zr | 23.4 | 72.1 | 55 | 1.01 | 1.73 | 29.4 | 66.3 |  | <1 | <1 | <1 | <1 | <1 | 1.21 | <1 | <1 | <1 | <1 | <1 | <1 |

**Table S2.** Major (Wt%) and trace (mg kg^‒1^) element contents of solid samples in the Bam salt plug.^a^

^a^ From ICP-MS analysis. Values for the major elements in Wt%, values for the trace elements in mg kg^‒1^ ^b^ bit: Bittern pond. Samples **Bit1** to **Bit4**: from center to margin of the Bittern pond. ^c^ Samples **12-1** to **12-4**: from center to margin of pond 12. ^d^ Samples **10-1** to **10-4**: from center to margin of pond 10.

| **Sample name** | **Li** | **B** | **Sr** | **K** | **Mg** | **Ca** | **S** | **Na** | **Cl** |
| --- | --- | --- | --- | --- | --- | --- | --- | --- | --- |
| base pond |  |  |  |  |  |  |  |  |  |
| **bp** | 1.0 | 1.0 | 1.0 | 1.0 | 1.0 | 1.0 | 1.0 | 1.0 | 1.0 |
| pond 10 |  |  |  |  |  |  |  |  |  |
| **t1** | 1.9 | 1.0 | 2.2 | 1.8 | 1.9 | 1.8 | 1.8 | 1.3 | 1.6 |
| **t2** | 4.6 | 1.0 | 3.7 | 4.3 | 4.3 | 1.3 | 1.6 | 1.0 | 1.2 |
| **t3** | 4.6 | 1.0 | 2.1 | 4.3 | 4.3 | 0.8 | 1.2 | 0.8 | 0.9 |
| **t3-I** | 9.4 | 1.0 | 4.3 | 9.5 | 8.8 | 1.6 | 2.3 | 1.3 | 1.4 |
| **t4** | 9.9 | 1.0 | 2.3 | 8.8 | 9.4 | 1.0 | 1.9 | 0.9 | 1.2 |
| **t4-I** | 14.0 | 1.0 | 1.4 | 12.4 | 13.6 | 0.8 | 2.2 | 0.9 | 1.2 |
| **t5** | 17.5 | 1.0 | 2.5 | 10.8 | 11.9 | 0.8 | 2.1 | 0.9 | 1.2 |
| Bittern pond |  |  |  |  |  |  |  |  |  |
| **bit-A** | 3.9 | 1.0 | 1.5 | 5.8 | 3.9 | 1.0 | 1.3 | 1.1 | 0.7 |
| **bit-A-I** | 5.5 | 1.0 | 2.0 | 4.9 | 5.0 | 1.2 | 1.7 | 1.3 | 1.3 |
| **bit-B** | 9.9 | 1.0 | 2.7 | 9.1 | 9.3 | 1.1 | 2.0 | 1.1 | 1.4 |
| **bit-B-I** | 7.2 | 1.0 | 2.0 | 6.6 | 6.8 | 0.7 | 1.4 | 0.6 | 0.8 |
| **bit-C** | 12.5 | 1.0 | 2.8 | 11.4 | 12.1 | 0.9 | 2.2 | 1.0 | 1.3 |
| **bit-C-I** | 19.5 | 1.0 | 1.7 | 8.0 | 9.2 | 0.5 | 1.6 | 0.7 | 0.9 |

**Table S3.** Boron-normalized value (NB) changes in the ponds of the Bam salt plug.
